# Understanding cultural perceptions of sexuality in China and their influence on human papillomavirus vaccine hesitancy

**DOI:** 10.3389/fpubh.2024.1462722

**Published:** 2025-01-23

**Authors:** Jiayuan Jin, Yanchao Han

**Affiliations:** School of Humanities, Donghua University, Shanghai, China

**Keywords:** human papillomavirus, vaccine, sexuality, hesitancy, culture

## Abstract

**Introduction:**

The human papillomavirus (HPV) vaccine prevents infections that may result in precancerous lesions, cervical cancers, genital warts, and many other diseases. However, its uptake rate in China is 2.24%, and HPV vaccine hesitancy is widespread. This study aimed to identify whether cultural perceptions of sexuality in China influence HPV vaccine hesitancy and explore the correlation between sexual attitudes, sexual morality, and HPV hesitancy in Chinese.

**Methods:**

Between January and April 2024, we adopted a qualitative approach and conducted in-depth, one-on-one, semi-structured interviews with 15 young female Chinese university students of sexual maturity.

**Results:**

Although all college students exhibited vaccine hesitancy, their perceptions of vaccines and reasons for hesitation varied. This study classified hesitancy into three types: individuals hesitant owing to cultural conservatism and stigmatization, individuals with positive intentions but delayed vaccination, and overconfident individuals who disregarded vaccines. The reasons for vaccine hesitancy are linked to China’s conservative culture of sexuality, and Chinese sexual attitudes and morality have complex mechanisms that influence vaccination. Social taboos on sexual topics lead to inadequate sources of information regarding the HPV vaccine. Sexual morality is internalized into an individual’s subconscious, influencing the cognition of the vaccine. Negative associations and stigmatization of the vaccine further discourage vaccination.

**Discussion:**

Vaccine hesitancy is not solely a medical or political-economic issue but also a deep-seated cultural issue. Culture significantly affects individual health behaviors, and ignoring socio-cultural factors can exacerbate public health problems. Future research should investigate the political-economic and socio-cultural factors to address geographic differences in vaccine hesitancy.

## 1 Introduction

Vaccine hesitancy refers to a delay in accepting or refusing vaccines despite their availability (1). In 2019, the World Health Organization ranked vaccine hesitancy among the top 10 global health threats. Vaccine hesitancy leads to decreased vaccine coverage and an increased risk of vaccine-preventable disease outbreaks and epidemics (2). Most studies on vaccine hesitancy have focused primarily on the medical field and assumed that it is caused by limited or a lack of biomedical information (3–5). The vaccination rate could increase if medical facts regarding the benefits and value of vaccines are well understood. Studies in this field have encountered two major challenges. First, medical knowledge alone does not necessarily lead to vaccine uptake (6). Vaccine hesitancy is also affected by socio-cultural and politico-economic factors, including the political environment (7), policy impacts (8), religious beliefs (9), media (10), and social norms and values (11). However, these social and political factors affecting vaccination are sidelined in understanding and responding to vaccine hesitancy (12, 13). Second, vaccine hesitancy varies significantly across places, populations, times, and even vaccines (6). Significant differences were observed in vaccine hesitancy between high-income and low- and middle-income countries (14, 15), among different cultural backgrounds (16), among ethnic groups (17), and over time for the same vaccine (18). However, current research on vaccine hesitancy mostly discusses interventions and neglects to emphasize diversity across different locations, populations, and periods. Despite these challenges, some studies have effectively considered socio-cultural and politico-economic factors, particularly in research on human papillomavirus (HPV) vaccine hesitancy. For instance, In the Global South, research highlights additional context-specific issues like access, which are not covered by the WHO’s definition of vaccine hesitancy. Cultural and religious beliefs, as well as reactions to government actions, also play a significant role in shaping vaccine hesitancy (19). Similarly, studies from other regions provide further insights. In Europe, Studies have explored factors influencing HPV vaccine hesitancy, focusing on public mistrust and uncertainty (20), and in Asia, where multidimensional social and cultural norms play a significant role (21). Meanwhile, In the United States, research has examined the role of race, ethnicity, and income in HPV vaccination rates (22). For example, vaccine hesitancy in multiethnic communities in Los Angeles has been influenced by historical mistrust, such as forced displacement in AI/AN communities, as well as societal barriers like transportation and work schedules (73). Despite these contributions, the focus has been on differences in vaccine hesitancy from a regional perspective, such as in Europe and Asia. Research on HPV vaccine hesitance across ethnic groups, countries, and regions is lacking.

HPV is often pathologically described as a genital wart, with sexual transmission as the primary route of transmission. Sex inevitably becomes a central topic in discussions about the HPV vaccine. Studies have discussed HPV vaccine hesitation as uniquely linked to adolescence, sexuality, and attitude toward sex and sexual practices (23, 24). Some studies have suggested that cultural and religious sensitivities pose a threat to HPV vaccination in Asia because of the predominant sexual transmission of HPV (21). Under patriarchal value systems, HPV is presented in a “feminized” and “moralized” manner, reinforcing the burden of disease on women (25). China faces similar challenges in HPV vaccination. On one hand, Socio-cultural sexual norms in China are complex. The recognition, dissemination, and uptake of vaccines are influenced by complex sexual attitudes and moralities. Any form of sexual practice in China had to comply with multiple sexual ethics, such as the taboo of sexual social communication, the primary goal of sexual reproduction, and the gender role norms of male superiority and female inferiority (26). On the other hand, the vaccination rate in China remains low, exposing women—particularly college students—to significant risks. Although Chinese attitudes toward sexuality have gradually opened up with economic and cultural development, traditional sexual norms remain deeply rooted and continue to shape public opinion, control and influence ideological activities, and influence social expression. According to the 2023 ICO/IARC report, approximately 98% of cervical cancer cases in Chinese women are caused by high-risk HPV strains. The incidence of cervical cancer has been increasing, ranking third in both incidence and mortality among women aged 15–44 in 2020. Female Chinese college students, particularly those aged 17–24, face the highest risk of HPV infection during adolescence and early sexual activity. A multi-center population-based study revealed that this age group exhibits the highest infection rates, with a notable “double peak” age distribution (75).

This study aimed to identify whether cultural perceptions of sexuality in China influence HPV vaccine hesitancy and explore the correlation between sexual attitudes, sexual morality, and HPV hesitancy.

## 2 Materials and methods

This study used qualitative methods. Individual, semi-structured, in-depth interviews were conducted with 15 female Chinese college students. Data saturation was achieved, and no new themes or codes emerged, confirming the validity of the themes and findings (27).

### 2.1 Data collection

Participants were recruited between January and April 2024 via purposive sampling with the following criteria: (1) aged 18–26 years, representing a sexually active age group at high risk of HPV infection. (2) Female college students with similar educational backgrounds and social environments. (3) Unvaccinated for HPV to ensure an in-depth understanding of vaccine hesitancy. (4) Currently hesitant about the HPV vaccine to focus on factors that influence vaccine acceptance. Special attention was given to ensure participants were diverse and represented vaccine hesitancy across different regions and majors.

The interviews were conducted in quiet, comfortable settings, such as the local Centers for Disease Control and Prevention or interviewees’ residences, to ensure maximum privacy and confidentiality. The participants provided informed consent and recording permission before the interviews. Participants were allowed to withdraw from the interview at any time, and all interviews were conducted anonymously. Each participant was assigned a code and pseudonym for the data. At the end of the interview, participants were compensated with 50–150 RMB (6.56–19.67 EUR). To protect privacy further, all audio files were destroyed after transcription and accuracy checks.

### 2.2 Statistical analysis

After a rigorous recruitment and selection process, we interviewed 15 participants (28). Personal information was collected from the interviewees (Table 1).

**Table 1:** Participant demographics.

Participant number	Age	Region	Education level	Vaccine hesitancy
A1	21	Chongqing Municipality	Senior	No infection path and low risk, unnecessary
A2	20	Hebei Province	Junior	Acknowledges vaccine but delays vaccination
A3	23	Henan Province	Graduate year 1	No strong desire to vaccinate, inclined to refuse
A4	24	Shaanxi Province	Graduate year 3	Acknowledges vaccine but delays vaccination
A5	23	Xinjiang Uygur Autonomous Region	Senior	Acknowledges vaccine but delays vaccination
A6	24	Shandong Province	Graduate year 3	Acknowledges vaccine but delays vaccination
A7	23	Guangdong Province	Senior	Unaware of vaccine, considers it unnecessary
A8	23	Zhejiang Province	Senior	High hesitancy, inclined not to vaccinate
A9	23	Jiangsu province	Graduate year 3	High hesitancy, aware of benefits but delays
A10	21	Shanghai Municipality	Senior	Acknowledges vaccine but delays vaccination
A11	24	Zhejiang Province	Graduate year 1	Wants to vaccinate, but will do so later
A12	22	Chongqing Municipality	Senior	considers it unnecessary
A13	23	Shandong Province	Graduate year 2	Unnecessary, will not vaccinate soon
A14	22	Henan Province	Graduate year 2	Does not want to vaccinate, inclined to refuse
A15	20	Jilin Province	Sophomore	Hesitant but inclined not to vaccinate

Any significant behavior exhibited by the interviewee during the interview was promptly recorded in a memorandum. After each interview, the recordings were transcribed verbatim and checked for non-verbal cues such as laughter, sighs, and situational information. This approach ensured a comprehensive understanding of the recordings, enriching the depth of the analysis. Any gaps or uncertainties in the recordings were addressed by contacting the interviewees. Audio recordings, memorandums, and interview transcripts played vital roles in providing a reliable review trial for the study. Subsequently, using thematic analysis (29) and NVIVO12 software, we coded, categorized, and thematically analyzed the interview data. Data related to the research questions were coded using a semantic approach to obtain a concise overview of the recurring points. Next, we identified the subthemes within these codes and reviewed them to ensure that they were accurate representations of the data. To strengthen the validity of the qualitative analysis, two professionals reviewed the thematic analysis at each stage, helping refine the subthemes determined for the study. Subsequently, we conceptualized themes within these subthemes and conducted a qualitative analysis. Data saturation was reached as redundant data indicated that further interviews would not generate new themes or codes (27). Interview excerpts were included in the codebook to ensure codes were based on the interview data. Throughout the analysis, we cross-checked interview excerpts, themes, and categories to maintain consistency and validity. This study followed the Consolidates Criteria for Reporting Qualitative Research (30).

## 3 Results

### 3.1 Status of vaccine hesitancy among female Chinese college students

During the interviews, all 15 female Chinese college students displayed vaccine hesitancy; however, the extent of hesitation varied significantly. This variation primarily stems from differences in the awareness of vaccines and underlying reasons for hesitation, which are influenced by factors such as sexual attitudes, sexual morality, and stigmatization. Perceptions of the HPV vaccine were shaped more by subjective awareness than by medical knowledge, as individuals often interpret information through personal or cultural lenses rather than its objective essence (31). The health behaviors of young adults, especially college students, whose values are still forming, are more influenced by social norms and people around them. Social norms and the attitudes of those around them serve as critical reference points for health-related decisions, especially when faced with new information (32, 33). As a result, subjective perceptions and understanding of the HPV vaccine vary significantly owing to differences in their sexual attitudes or sexual morality. Individual vaccine hesitancy has a unique position in the spectrum, ranging from complete acceptance to outright rejection. According to the classification used in this study, the 15 respondents were divided into three groups: Individuals are hesitant owing to cultural conservatism and stigmatization, Individuals with positive intentions but delay vaccination, and overconfident individuals who disregard vaccines.

#### 3.1.1 Individuals are hesitant owing to cultural conservatism and stigmatization

Four respondents exhibited hesitation toward vaccination, primarily owing to culturally conservative attitudes toward sexuality and the stigmatization associated with HPV vaccines. In Chinese society, where discussions of sexuality are often considered taboo, HPV—closely tied to sexual health—faces similar avoidance in public discourse. This cultural dynamic limits access to accurate vaccine information and reinforces hesitancy.

A15: It is not easy to explain to the family that they will only be more pedantic than my friend’s kind of thinking, which is quite prejudiced against women’s diseases. It’s not something we talk about at home. My parents avoid these topics entirely, so I’ve never had a chance to learn much about it or ask questions.

These individuals are also affected by the stigmatization of viruses and vaccines. Stereotypes of sexuality, negative associations, and stigmatization of diseases still exist in Chinese societies, triggering college students’ rejection of vaccines. Some interviewees did not go for vaccinations to prevent discrimination and prejudice.

A1: I heard classmates discussing that someone who got the HPV vaccine later engaged in casual sexual relationships. I fear my classmates or even my parents might misunderstand, assuming that I’ve already been sexually active or am promiscuous, so I’m hesitant to get vaccinated.

#### 3.1.2 Individuals with positive intentions but delay vaccination

During the interviews, most participants expressed ambivalence toward vaccination. They acknowledged the benefits of the vaccine, such as reducing the risk of cervical cancer infection and preventing genital warts; however, they hesitated due to the limited availability of reliable information, which is shaped by cultural factors surrounding sexuality. The prevalence of negative rumors and lack of reliable information sources left them uncertain about the vaccine’s safety and efficacy. Some respondents also underestimated their risk of infection, misconstrued the priority of the vaccine, and delayed vaccination. This hesitancy reflects a broader cultural context in China, where conservative attitudes toward sexuality restrict access to reliable vaccination information. Because of privacy concerns, individuals are less likely to discuss sexual health, including vaccines related to sexually transmitted infections, leading to an information gap. The scarcity of information makes them more vulnerable to misinformation, such as the side effects and safety of vaccines, on social media and unofficial medical accounts, making them overly concerned about the negative reports of vaccines and amplifying their fear of the unknown risks of vaccination.

A2: Vaccination is protective; however, I am more concerned about its side effects. For instance, after receiving a previous vaccine, I still contracted the illness and experienced side effects. I’ve also come across posts online suggesting that the HPV vaccine causes severe side effects, which makes me very anxious and unsure about getting vaccinated.

A3: I am generally aware of the benefits of the vaccine, but I do not feel any urgency to get vaccinated. I thought it was something I could do at any time without age restrictions. I have not come across many official or detailed articles on this—most of what I know is from occasional posts on social media.

#### 3.1.3 Overconfident individuals who disregard vaccines

In addition to the previously discussed categories, four college students considered vaccination unnecessary and had an indifferent attitude toward it. This attitude appears to be influenced by beliefs surrounding premarital sexual behavior and traditional views on female chastity. They believe they are at a lower risk of contracting HPV or developing cervical cancer and think they are capable of coping with HPV through preventive measures, such as avoiding high-risk sexual behaviors or undergoing regular screening. Moreover, some female students expressed that without engaging in sexual activity, vaccination seemed irrelevant. This view aligns with the cultural belief that abstinence or limited sexual exposure negates the need for preventive measures like vaccination.

A1: I am relatively young and not in a vulnerable age group. I believe that the risk of contracting these viruses is low. Many of these viruses are transmitted through sexual intercourse, which is not a concern. Therefore, I do not think there is a need to be vaccinated. If I practice good hygiene, pay attention to sexual health, and avoid overly revealing clothing outdoors, I feel protected.

A14: I honestly feel no need to get vaccinated. This vaccine was introduced from abroad, but generally, Chinese people are conservative about sex. Since I do not engage in sexual activity, I see no reason to get vaccinated—perhaps only after marriage, if necessary.

### 3.2 Culture of sexuality influences HPV vaccine hesitancy: sexual attitudes, sexual morality, and stigmatization

The vaccine hesitancy of the female Chinese college students was mainly influenced by sexual attitudes, sexual morality, and stigmatization, with the following three influencing mechanisms: Social taboos on sexual topics lead to inadequate sources of information regarding the HPV vaccine. Sexual morality is internalized into an individual’s subconscious, influencing the cognition of the vaccine. Negative associations and stigmatization of the vaccine further discourage vaccination. These mechanisms are deeply rooted in the cultural context, where traditional Chinese feudal rites and rituals continue to exert a significant influence on sexual attitudes. This influence is not only affects people’s thoughts but also subtly penetrates everyone’s values, shaping perceptions and behaviors toward sexuality and vaccination. Katchadourian and Lunde (69)reported that understanding the historical origins of cultural attitudes toward sex is crucial for understanding current behaviors. Historically, Chinese sexuality was influenced by Taoist concepts, particularly the yin-yang duality, wherein sexual activities symbolized the harmony between yin and yang, essential for universal balance (34–36). Within Taoism, sexual expression was not inherently discouraged; rather, it was embraced as a natural interaction essential for cosmic balance (76). However, this open attitude toward sexuality shifted dramatically following ideological changes during the Song and Yuan dynasties. During the Song and Yuan dynasties, the representative of feudal ritualism, Cheng-Zhu, advocated feudal ethics and asceticism in all societies. Sex became a taboo topic limited to public discussions, and views of chastity developed further. The strengthening of feudalism in the Ming and Qing dynasties led to a further decline in the status of women and restrictions on their sexual rights (37). Confucian teachings reinforced these constraints by urging the repression of desires, particularly sexual impulses, to maintain social order (71). This framework promoted female virginity as vital to family honor and lineage purity (36). Consequently, premarital sex was strictly prohibited, while extramarital affairs were severely penalized (77). In modern times, exposure to Western views has somewhat liberalized sexual attitudes, loosening traditional moral expectations, such as chastity and fidelity, with premarital sex no longer regarded as moral transgression for women (38). However, cultural inertia persists, with ideological and societal perceptions lagging behind material changes (39, 40, 72). Patriarchal structures and the revival of traditional familial culture, especially with Confucianism’s reintroduction in the 21st century, have perpetuated entrenched beliefs (41). This historical and cultural legacy continues to influence HPV vaccine hesitancy, even among well-educated female college students. Despite their higher levels of education, these students still confront shame surrounding sexuality, adherence to chastity and premarital norms, and stereotypical gender norms. These factors collectively contribute to a cautious approach to HPV vaccination, reflecting the ongoing impact of traditional cultural perspectives.

#### 3.2.1 Social taboos on sexual topics lead to inadequate sources of information regarding the HPV vaccine

The social climate of embarrassment surrounding discussions about sexuality creates significant barriers to communication regarding sex-related diseases, particularly HPV. This atmosphere of shame creates obstacles to accessing accurate information about HPV vaccines, contributing to vaccine hesitancy. Shame plays an important role in Chinese culture (42). Sexuality is often regarded as taboo, leading to what is commonly referred to as “sexual shyness.” This cultural phenomenon is deeply rooted in traditional Chinese sexual culture, where open discussions about sex are considered inappropriate, and the expression of sex is implicit, ambiguous, and evasive. Topics like mental illness and sexually transmitted diseases are similarly stigmatized topics, often viewed as sources of disgrace in Chinese society (43). This ingrained shame largely stems from concerns over mianzi and is compounded by prevalent body shame and body dissatisfaction, factors that contribute to heightened sexual shame among Chinese adults (44). As a result, this atmosphere of shame creates obstacles to accessing accurate information about HPV vaccines, contributing to vaccine hesitancy.

##### 3.2.1.1 Taboos on sexual topics in Chinese families

The influence of family on female college students is significant yet subtle, especially regarding discussions about sexual health. In many Chinese families, taboos on sexual topics are deeply ingrained, resulting in a lack of sexual health education within families. This sexual attitude prevents families from effectively conveying important information regarding vaccines. Family education has the unique advantages of blood sentiments and the continuity of parents’ education of their children. However, these advantages do not extend to the transmission of knowledge on sexual and reproductive health. Talking about sex is a taboo in Chinese families, especially among children. Parents do not talk about it to their children, and children never dare to bring it up (78). Evidence suggests that even when parents do provide some sexual education, the content remains limited and narrow in scope (45–48). Such education often relies on a one-directional approach, emphasizing control or warnings rather than fostering open discussions. For many parents of today’s college students, born during the 1960s and 1970s, discussing sexual matters was even more stigmatized. Consequently, many of these parents lack adequate sexual education and may be reluctant or unsure about addressing their children’s questions. When they do broach these subjects, they often use simplistic, vague, or indirect language (49). Research indicates that even parents with some sexual health knowledge rarely engage in sexual discussions with their children due to cultural or religious constraints, resulting in low communication frequency (50). During the interviews, college students said they did not know how to talk to their parents about the HPV vaccine, which is highly correlated with sexually transmitted diseases, and used the words “embarrassed” or “ashamed” to describe conversations with their parents about the HPV vaccine, or worried that their parents would be suspicious of their motives for vaccinating themselves against sexually transmitted diseases.

A5: My family did not know about the vaccine, and even if they knew that it could combat sexually transmitted diseases, they would be skeptical. In particular, I do not know what to tell them, and I am probably embarrassed to tell my parents that it is preventing this disease. Talking about sex with my parents will be awkward, and I have not talked about it before.

A13: My parents never talked to me about this vaccine, even though some people around me have mentioned it. The atmosphere at home is still quite repressive. From a young age, they avoided exposing me to anything related to sexuality. They’ve never mentioned things like this, let alone vaccines.

##### 3.2.1.2 Sexuality viewed as a private matter in interpersonal relationships

Peer communication is an important method for college students to acquire information, especially among those of similar age and experience. However, sex is regarded as a private matter in traditional Chinese culture, restricting open communication on sexually sensitive issues. Behavioral interaction between peer groups and mutual influence can positively impact behavior and attitudes, fostering self-growth and collective development (68). Peers sharing knowledge and exchanging ideas should effectively disseminate knowledge about HPV vaccines. However, social shame and taboos surrounding sex, viewed as a private, individual matter, impede open discussions (70). Consequently, social networks among college students failed to provide an effective channel for disseminating HPV vaccine information. The interviews revealed that even when college students talked about the HPV vaccine, they focused only on vaccine appointments and vaccination experiences, avoiding topics related to sexual transmission.

A3: I have hardly ever heard discussions on sex and sexually transmitted diseases offline. People seldom discuss these topics seriously. Although menstrual shame is not advocated for, talking about sex naturally is still not widely accepted. Moreover, among friends, people do not have a deep relationship to discuss their sexual behavior. With my classmates, we do not talk about whether or not we have sex or whether we need to get vaccinated.

##### 3.2.1.3 Implicit expressions of sexuality in social communication

Owing to the taboos surrounding sexual topics, the implicit expression of sexuality in mass media results in a lack of awareness of the HPV vaccine. Mass media, a key conduit of social communication, contributes to the limited coverage of HPV and vaccines. Within family settings, “sexuality” remains largely unspoken, and in classrooms, discussions around sex are often viewed as awkward or inappropriate, pushing college students toward more open, mobile platforms for seeking sexual health knowledge (51). Current media trends, however, reflect inadequate reporting on HPV, with both traditional and new media channels failing to adequately communicate scientific knowledge about the virus and the vaccine ((52); Ren, 2010). Research shows that in mainland China, while the volume of HPV vaccine coverage in the media has gradually increased, it still remains at an “information” news stage. Reporting is predominantly positive, with a preference for government sources and topics centered on vaccine-related policies. Due to its association with sexuality, coverage on HPV by official media is often restricted, with explicit references to sexual health either avoided or obscured to the point of diminishing public awareness. During the interviews, college students indicated that although they had been exposed to much information about HPV from social networks, effective and scientific information was limited, especially from authoritative sources. Most online posts are from netizens, who mainly share their personal feelings, including the sharing of HPV vaccination experiences and concerns about the side effects of the HPV vaccine, which are not related to scientific knowledge of the HPV vaccine. Additionally, the official popularization of the vaccine often emphasizes its importance, neglecting crucial sex-related information, such as transmission pathways.

A12: If you ask where I get information on the HPV vaccine, I’d say it’s mostly from the internet, but mostly from posts by other users. I’ve rarely come across anything from official sources, so honestly, I do not fully understand this vaccine.

A7: I’ve noticed that official public accounts or science communication platforms do not post much about the HPV vaccine. Even when they do, the content is quite superficial. Most of the time, it’s just about encouraging us to get vaccinated without providing detailed or useful information.

Topic taboos hinder information dissemination about HPV and the vaccine across various levels, including family, peers, and society, resulting in a scarcity of “valuable” and “useful” information for the public (53). Pervasive taboos have resulted in insufficient and inaccurate vaccination information. Online content and posts frequently lack valid information on the virus and vaccination. The lack of knowledge about the safety of vaccines is reflected in interviews with college students who believe that vaccination involves a risk of side effects.

#### 3.2.2 Sexual morality is internalized into an individual’s subconscious, influencing the cognition of the vaccine

Sexual morality strictly prohibits female premarital sex and the social norms of female chastity are internalized in personal values, causing female college students to have biased perceptions of risk and benefit when perceiving sex-related illnesses, affecting the cognitive subject. The views of chastity that exploits and destroys women are not aligned, placing significant moral constraints on women (54). This social fear and moral oppression of losing chastity extends toward sexual diseases. Those infected with HPV are hesitant to disclose their infection publicly owing to shame and fear of facing social discrimination and moral criticism. However, only sex based on procreation is a ‘proper sex’ recognized by the mainstream culture of society (55). Moral norms prohibiting premarital sex and the perception of sexuality only within marriage and for procreation have affected women’s benefit perceptions. Over time, these beliefs are internalized from the external level of social and moral norms to the internal subconscious of women. They have influenced women’s perceptions and attitudes toward sexuality and inadvertently shaped their perceptions and behavioral choices regarding health issues such as sexually transmitted diseases and vaccines.

##### 3.2.2.1 Chastity norms and biased perceptions of HPV infection risk

The moral pressure on women to remain chaste contributes to silence around HPV infections, causing college students to misjudge disease susceptibility and severity, resulting in vaccine risk perception bias. Individuals form risk perceptions about specific health threats, and in the health decision-making process, they must weigh the potential consequences and benefits of actions to make a choice (67). In the context of health decision-making, these perceptions are crucial in shaping behavior However, in a cultural context where women’s morality is closely tied to chastity, sexually transmitted infection often incurs severe social and moral judgments, branding the affected individuals as morally compromised and promiscuous. Within patriarchal cultural norms, women are frequently expected to remain passive in sexual matters, while men face fewer restrictions on sexual expression, including polygamous or commercial engagements (41). This disparity reinforces societal expectations that stigmatize women while allowing men greater sexual freedom. Consequently, when women are infected with HPV, they often conceal their condition owing to fear of discrimination and prejudice to avoid being blamed by others. Silencing the infection leads to misjudgment of disease susceptibility and severity among university students. During the interviews, college students said that they seldom heard of HPV infections around them and that the chances of virus infection are low; therefore, precautions are not necessary. Additionally, since high-risk HPV infections are strongly associated with sexual activity, individuals who contract high-risk strains are often reluctant to disclose their condition. As a result, many college students also said that in an invisible network society, they have heard of the experiences of netizens infected and that their symptoms are not serious, and the treatment process is relatively easy; therefore, HPV infection is not significant and does not need to be taken seriously. This is in contrast to the actual rate of HPV infection and severity of cervical cancer, and the seriousness of HPV infection should not be underestimated.

A9: Honestly, I think it’s a bit of a fluke; I’ve grown up not hearing much about anyone around me getting this infection. From what I’ve seen online, most cases seem to be of the low-risk type, where a simple course of medication is enough. It does not seem nearly as severe as the reported statistics suggest, so I do not feel the need for vaccination.

##### 3.2.2.2 Limitations on premarital sexuality and diminished perception of vaccination benefits

Influenced by the morality of prohibiting premarital sex, female college students who generally do not have sexual partners believe that vaccination is not necessary, creating a temporal bias toward vaccination and further affecting the accuracy of the perceived benefits of vaccination. In Chinese societies, most people are cautious and conservative about premarital sex (45), and this moral norm shapes the sexual behavior and health choices of youths, especially female college students. Most Chinese youth still seek stable love and marriage relationships, and single female college students believe that they will not have a sexual partner in a short period, reducing the urgency of HPV vaccination, which leads to misjudgment about the timing of vaccination. In China, married women have a higher perceived benefit of HPV vaccination owing to their family roles and reproductive needs, and the role of HPV vaccination has changed from an emergency preventive measure to a health guarantee for maintaining long-term relationships (65). Young college students believe that sexuality is still far away, cancer is still a long way off, and that there is no need to panic and consider HPV vaccination prematurely. This “time bias” makes them think that the vaccine is important but not urgent, not realizing that the earlier they are vaccinated, the more protection they can get from the HPV vaccine, which is widely ignored (66).

A13: I do not have a boyfriend now, and sex is not necessary at the moment, right? Even though there is an optimal age to get vaccinated, and it is not completely ineffective past that age, even if I get vaccinated now, it does not mean I will never get it again. I know some of the benefits of vaccination; however, there is no point where I can push to get vaccinated, maybe in the future, when I am in a relationship and will have sex.

Perceived risks and benefits of HPV and vaccines are important aspects of the decision-making process, and sexual morality is internalized subconsciously, which are prevalent in the vaccination decision-making process. During the interviews, college students said, “I do not think I can get this disease; therefore, there is no need to get vaccinated,” and since” I do not have a sexual partner, why should I get vaccinated?” Opinions such as these are manifestations of cognitive biases that affect vaccination decisions and explain the factors that explain vaccine hesitancy among female Chinese college students.

#### 3.2.3 Negative associations and stigmatization of the vaccine further discourage vaccination

Sexual stereotypes in Chinese society have led to negative perceptions of HPV and its vaccine, often accompanied by moral criticism. These biases stem from deeply ingrained cultural norms and misconceptions about sexuality. Misinformation about the virus and its prevention further perpetuates the stigmatization of vaccinated women, particularly within a patriarchal social framework. This affects college students’ vaccination decisions and causes vaccine hesitation. Feudal and traditional cultures have ingrained sexual stereotypes in Chinese societies, which have not only given rise to prejudices and misconceptions about sexuality but have also given a negative association to sexually related diseases. Within the patriarchal discourse of traditional Chinese culture, moral bondage advocates that the sexes are not equal. Regarding sexuality, the same event or behavior is often evaluated significantly differently by men than by women. This double standard reinforces the stigmatization of female-related health issues, including those associated with HPV.

##### 3.2.3.1 Negative associations of HPV and vaccines

HPV, as a sexually transmitted virus, is deeply intertwined with perceptions of sexuality in China. Chinese stereotypes of sexuality have given rise to specific negative associations and moral critiques of HPV. In specific cultural contexts, all viruses or diseases create negative associations, influencing people’s perceptions and lives (74). The negative associations surrounding the disease have an impact that goes beyond the harm that the disease does to the individual or society, being moralized about the disease and further reinforcing prejudice. This negative association originates from the stigma and prejudice against sexual diseases in traditional Chinese sexual culture, and the stereotype of sex has led to an unlimited amplification of the sexual element in all sexual diseases (79). HPV, being mainly transmitted sexually, creates a labeled understanding of HPV, and the public believes that inoculation with HPV means paying for impure behaviors. During the interviews, this negative association was evident, with some respondents believing that a positive HPV result sent negative messages about stigma, impurities, and sluttiness. HPV infection is directly understood by some students as a “sexually transmitted disease,” and under the influence of sex prejudice, the HPV vaccine is regarded by some students as “exclusively for women” or “required for sexually active people,” which is further influenced by negative associations and stigmatization.

A6: I have not looked very deeply into HPV infection, and I always think that with these diseases, especially sexually transmitted diseases, the private life is not very clean; you do not pay attention to hygiene, or there could be some other reasons as well.

##### 3.2.3.2 Stigmatization of HPV vaccine recipients

The negative moral association of HPV further contributes to the stigmatization of those getting vaccinated. Women, in particular, face heightened discrimination due to the pervasive influence of patriarchal societal norms. In male-dominated systems, women are subjected to stricter moral scrutiny and often bear the brunt of stigma and social judgment related to sexually transmitted infections and their prevention. Stigma is a serious discrediting attribute that reduces a person from an ordinary individual to an insulted, defiled person (56). What is stigmatized in different contexts depends on what is considered the dominant cultural practice at that time. Individuals facing stigmatization are usually not accepted by the dominant cultural practices of their time context and are often subjected to injustices from society, leading to mental or even physical harm (57). Within traditional Chinese patriarchal culture, women are often held to higher moral standards than men, particularly regarding sexual behavior. This cultural expectation not only reinforces gender inequality but also amplifies the stigma associated with HPV vaccination.

A4: Throughout history, women have always been at a disadvantage. When women get the vaccine, they are often seen as sexually active or promiscuous, even if that’s not the case, and this reflects broader prejudices against women’s health issues. The truth is, many people who get HPV have not contracted it because of promiscuous behavior, yet they still end up with negative labels. It’s even possible that it was transmitted by men in the first place.

HPV vaccination prevents the disease. However, under the influence of male dominance over women, vaccination is given unnecessary moral overtones. Infection is perceived as a punishment for slutty behavior, and the vaccine recipient is associated with uncleanliness, a symbol of defilement. By associating HPV vaccine recipients with women’s sexuality, beliefs such as “only immoral women get the HPV vaccine” or “getting the HPV vaccine is an admission of sexual behavior” are widely circulated in society. During the interviews, HPV vaccine recipients were considered a sexually active group, and “only women with multiple sexual partners are at a high risk of HPV infection.” The notion that “it is all about intercourse” persists, and labels such as indiscretions or sexually explicit descriptions that are insulting and derogatory and words such as promiscuity that are stigmatizing are ubiquitous among HPV vaccine recipients. Gynecological diseases have nothing to do with women’s sexual morality, and this stigma ignores the scientific fact that women are more susceptible to sexually transmitted infections than men because of certain characteristics of the vaginal environment and potential tears during intercourse. Even with only one sexual partner, women may be exposed to their biology and have a higher risk of developing the disease (58).

A9: I have a classmate who got vaccinated, but I heard some peers gossiping afterward, saying that she felt like she did not care about the virus after getting vaccinated, and then had a very chaotic private life; it felt as if she could casually have sex because she was protected after finishing the vaccination.

HPV is a sexually transmitted disease virus that involves paying for unclean sexual behavior, and vaccine recipients are considered a group of people with chaotic private lives; this makes college students worry about receiving malicious speculations about motives or even stigmatization and avoiding the risks posed by the vaccine, resulting in vaccine hesitation. During the interviews, college students were concerned that their parents and friends would think they were having premature sexual behavior after being vaccinated. Some college students were influenced by the stigmatization of vaccine recipients and did not want to risk being misunderstood by getting vaccinated. This surrounding group pressure puts a strong psychological burden on vaccine recipients, causing college students to avoid the vaccines, and potential vaccine recipients do not want to be vaccinated based on “risk avoidance,” which influences college students’ vaccine decision-making and creates vaccine hesitancy.

## 4 Discussion

This study explored the cultural influences on HPV vaccine hesitancy among female Chinese college students, focusing on the impact of sexual attitudes and morality. Three categories of vaccine hesitancy among female Chinese college students were found: Individuals are hesitant owing to cultural conservatism and stigmatization, Individuals with positive intentions but delay vaccination, and overconfident individuals who disregard vaccines. Additionally, we examined three specific influencing mechanisms of sexual culture: Social taboos on sexual topics lead to inadequate sources of information regarding the HPV vaccine. Sexual morality is internalized into an individual’s subconscious, influencing the cognition of the vaccine. Negative associations and stigmatization of the vaccine further discourage vaccination.

First, the population was categorized according to the degree of vaccine hesitancy. While vaccine hesitancy has traditionally been categorized simply as “acceptance” or “refusal” of vaccination attitudes and behaviors (59), recent scholarship has offered more nuanced classifications. Scholars have re-categorized people with varying degrees of vaccine hesitancy, such as “acceptors,” “opponents,” and “hesitants” (60), or “acceptors,” “hesitators,” and “rejectors”. They are also categorized as “confident,” “formalist,” and “awakened” according to the cognitive process or trait to differentiate between the “risk perception process” and “health perception process”. However, these classifications remain insufficient, as individuals’ underlying perceptions of vaccines and the reasons that lead to them differ, and generalizing these populations lacks relevance. Our research on the division of vaccine-hesitant populations provides theoretical support for further research and individualized promotion measures for subsequent populations with different vaccine hesitancies.

Second, this study conducted an in-depth investigation of the influence mechanism of sexual concepts and morality on vaccine hesitation through an in-depth interview and analyzed three influence mechanisms based on the interviews. Most studies on HPV and vaccines explore audience awareness and attitudes, using limited research methodologies such as questionnaires (61) or discussing the hesitancy of domestic and international HPV vaccines generally (62), and most conduct qualitative studies on HPV vaccines to acquire data. Few studies have used qualitative methods to obtain data and draw conclusions. This study, in contrast, explores how sexual cultures influence vaccine hesitancy using qualitative methods. Sexual shame creates taboos on sexual topics through the family, interpersonal, and social dimensions, causing female college students to lack knowledge about vaccines, resulting in vaccine hesitancy. The concept of female chastity makes female-related sexual diseases face a stronger moral critique, and this critique makes the infected remain silent, causing a misjudgment of vaccine susceptibility and severity. Perceptions of premarital sex affect women’s perceptions of diseases, such as HPV infection, and lead to biases about the effectiveness of the vaccine, further affecting vaccine hesitancy. Stereotypes of sexuality and discrimination against women under patriarchal society create negative associations and stigmatization of vaccines. The interviewees chose to avoid vaccines because of social and moral pressures and risk avoidance, leading to vaccine hesitancy.

Third, future research should incorporate more socio-cultural perspectives. The ecological systems theory posits that an individual’s development is influenced by several interconnected environmental systems, from immediate surroundings (family) to broad societal structures (culture) (63). An individual’s self-growth, social development, or health behavior following HPV vaccination is influenced by the traditions and moral norms of a society’s culture. Vaccine hesitancy extends beyond policy or isolated medical concerns, being deeply rooted in socio-cultural traditions, moral norms, and collective perceptions. Addressing this complex issue effectively requires an approach that incorporates local power structures, political contexts, and specific societal norms, enabling public health strategies to reflect the unique dynamics within each community. Additionally, studies of health behavior should transcend a purely biomedical perspective, which can limit understanding. Medicine is inherently linked to cultural contexts, and the shift from a biomedical model to a biopsychosocial one highlights the need for a holistic view of individuals (64). Consequently, health issues intertwined with social pathologies must be analyzed within their specific social and cultural frameworks, an area in which research remains limited. The “problematization” of health behaviors as social and cultural phenomena is as significant, if not more so, than their biomedical aspects.

Based on our findings, the following recommendations are provided for vaccine hesitancy research and the public health field. Healthcare practitioners should adopt multi-channel outreach efforts to eliminate sexual shame associated with HPV and promote an open, accurate discourse on sexual health. Practical steps include implementing comprehensive awareness programs to dispel sexual shame by fostering a culturally sensitive educational atmosphere for sexual health, both online and offline. Community and school activities can also play a vital role by engaging participants in group discussions or educational activities that aim to destigmatize sexual health topics and promote vaccine acceptance. Additionally, initiatives specifically focused on stigma reduction are essential, actively addressing and reframing the narrative around HPV and similar vaccines to encourage preventive health without moral judgment. Further research is urgently needed to examine the cultural and social dimensions influencing health behaviors. Future studies could benefit from integrating medical anthropology, employing both qualitative and quantitative approaches, and juxtaposing modern and traditional views to reveal insights on health perceptions across mainstream and marginalized communities. Moreover, expanding the sample size to include diverse colleges and regions in China would enhance the generalizability of findings. Incorporating perspectives from healthcare workers administering HPV vaccines could provide valuable insights into frontline experiences, helping to tailor vaccination efforts in a manner that resonates with the population they serve.

This study provides a comprehensive examination of how Chinese culture of sexuality influences the current status of vaccine hesitancy among female Chinese college students, revealing the impact of a unique socio-cultural context on the health behaviors of a population in a particular region. Sexual shame, norms of premarital sex, chastity, and sex stereotypes are deep-seated social and sexual morality concepts affecting the information source and cognitive subject of vaccine awareness, forming a barrier to accessing information about the HPV vaccine, leading to limited cognitive understanding of HPV vaccine among college students, affecting the decision-making process, and causing vaccine hesitancy. In the field of public health, health behavioral issues due to social background need to be considered, as each place has a different religious, political, and cultural background, which influences people’s behavioral choices. The mechanism of this influence is also more complex, requiring more geographic qualitative research in the field of public health in the future.

This qualitative study provides valuable insights into the cultural influences on HPV vaccine hesitancy among female Chinese college students. However, several limitations must be considered. Firstly, the study’s sample size was constrained by limited time, resources, and personnel, potentially compromising the reliability of the findings. Moreover, the exclusive focus on a specific demographic—female college students from a single region in China—may restrict the generalizability of the results to broader populations and geographic locations. Cultural variations across different regions within China could further influence vaccine hesitancy differently, emphasizing the need for caution when extrapolating findings beyond the study’s specific context. Additionally, qualitative methods used in this study, while conducive to exploring nuanced cultural influences, are inherently subjective and susceptible to researcher bias. Future research should incorporate diverse participant groups and employ methodological triangulation to enhance the robustness and applicability of the findings across varied cultural and demographic settings.

## Data Availability

The original contributions presented in the study are included in the Supplementary material. Due to privacy concerns, the data is not publicly available. For further inquiries or access to the data, please contact the corresponding author.
