# Supplementary material for: Understanding cultural perceptions of sexuality in China and their influence on human papillomavirus vaccine hesitancy
Source: Front Public Health. 2025 Jan 23;12:1462722. doi: 10.3389/fpubh.2024.1462722 (PMC11801254; doi:10.3389/fpubh.2024.1462722)
Supplement: Supplementary file 1 [file Data_Sheet_1.zip › Frontiers_Supplementary_Material/Interview Transcripts - Participant 1.docx]

**Interview Transcripts - Participant 1**

A: Hello, first of all, I'd like to ask, what information do you currently have about the HPV vaccine?

B: Well, from what I understand, there are different types of vaccines available: bivalent, quadrivalent, and nonavalent, both domestic and imported. Each type of vaccine functions differently; higher-tier vaccines seem to protect against more diseases or viruses. There's also a lot of information online about the vaccine causing various side effects and debates about whether it's effective, especially for young women.

A: Alright, based on the information you know about the vaccine, how high is your current willingness to get vaccinated? Do you have any concerns or hesitations about it?

B: My willingness is moderate. The cost is relatively high, and I hesitate because I'm still young and don't feel at risk of contracting these viruses. So, I don't see a strong need to spend the money. Many of these diseases are primarily transmitted through sexual activity, and I don't have concerns in that area.

A: So, your current hesitations are mainly due to the cost being high and feeling young and not at risk. Besides these two points, do you have any other concerns about the vaccine?

B: Another concern is potential side effects. I read some alarming posts online a while ago about vomiting and developing new diseases like urticaria. Some comments by self-proclaimed medical students or knowledgeable individuals make it seem like the vaccine is ineffective or that domestically produced vaccines are unsafe. These comments receive a lot of likes and appear quite professional, which further discourages me from getting vaccinated.

A: You mentioned concerns in the comments about the safety of domestically produced vaccines. Are you personally concerned about the safety of domestically produced vaccines if you were to choose to get vaccinated?

B: Yes, I have some concerns because whether it's four shots or three shots, each dose could potentially have issues. I remember when the nonavalent vaccine came out, it wasn't widely accepted initially, with many saying the technology wasn't mature enough. It's risky; if something goes wrong, it could be quite serious and not worth the risk.

A: Alright, combining your previous answers, I have a general understanding of your concerns and hesitations about the vaccine. Given that you mentioned your willingness is moderate and you have some vaccine hesitations, what factors might prompt you to register for the HPV vaccine in the future? For example, as you mentioned earlier, you might need to observe for a period, see if the HPV vaccine matures, or other factors.

B: Well, in the next year or two, if I understand more about this vaccine, I might consider it once I have stable employment and income. Also, when my partner life becomes more settled, probably around 24 or 25 years old, I'll assess the maturity of the technology and definitely check online reviews before making a decision.

A: Well, you just mentioned in the concerns about getting vaccines and vaccines, well, your partner's life, so you don't have to discuss it, which means you think your partner's life follows fighting. Some of the relationships between the HPV vaccines.

B: Well, as far as I know, one of his ways of transmission is this sex life, so he thinks this disease. It's a big relationship, and having a partner is a must, right, even if there will be a partner and a sexual life. But I don't think there is a necessary relationship between the two, not to say that both vaccinated, may be that kind of life is not too serious. Don't speculate about others, well, because maybe others just want to better protect themselves, just choose different things. Because this disease is not a girl can decide, maybe the other side will hide, so this is just a means of self-protection, so like me to guarantee themselves. There is no possibility of this behavior, just not playing is a situation, so you have this experience, you can ensure their own safety, you can not play. Anyway, with this sexual stigma, I think it is not, don't think so.

A: Do you think that if you're going to get A nine-price vaccine and go to the gynecology department, will you ask if you have sexual experience, well, you will accept it normally, or may you feel some embarrassed?

B: Because I haven't experienced it yet, I would feel a little embarrassed, at my age. However, when wearing a mask and I don't know me anyway, I will feel a little embarrassed psychologically, but I will certainly be more comfortable, and I will feel that this is a normal way to ask.

A: well, good good, that just want to you to advance with prompted vaccine, prompted to play vaccine point had A more complete understanding, and extend out some small problems, well, in the end, we mainly is to discuss some, access to information, and whether like others science, first of all, you just mentioned you will be through the network to understand the HPV vaccine information. In the HPV information on the Internet you know, do you think it is a positive factor, more positive information, or negative, such as fake vaccine, the vaccine has more side effects, or is it relatively neutral?

B: It should be relatively neutral, with both the good and the bad ones, but people will have a good evaluation of the bad and a psychological bias. Will pay more attention to the existence of bad factors, which will affect their own decisions.

A: In addition to some negative factors on the Internet, there are some other things, such as grabbing seedlings, and then having to queue up for two or three years. Will you be affected by these posts?

B: Yes, if small places don't use some connections, they can't buy this quota. There will be a lottery in big cities, and then in small places I feel that a lot of classmates around me have given this vaccine through the familiar people at home.

A: You just mentioned your classmates. In addition to the online approach, have you ever talked to your classmates offline about HPV vaccine related information? In the communication with my classmates, what information do they convey more? For example, if I get the vaccine through the relationship, you also go to play, or say, I have some concerns about the vaccine is more positive or negative?

B: Well, after talking, they are usually from the family and ask them to fight, so they find connections to fight them. In fact, they have no negative feelings, generally think that this is a very. Need the thing, and also very normal thing, well, go to play, this vaccine is very very common a thing, with play what hepatitis B ah, this kind of vaccine is also similar, just play a protective effect anyway, they also won't worry too much. Although their family wants them to fight, they must be eager and willing to play.

A: Well, you just mentioned the family factor, have you ever asked you to get the HPV vaccine, and then they have any opinions on the vaccine, or some opinions.

B: They are supportive, but I don't think that much, so I respect my wishes. In fact, they also see those information on the Internet, on the marketing number issued, say that blow these vaccines more than many good, what this disease or this disease infection. The girl ah, all want to hit this, with these marketing number will also affect.

A: Our last question is, well, except if you are influenced by others, have you ever popularized others, or helped others solve some HP vaccines. The question is if anyone asked you about the HPV vaccine, and then how did you answer them, or how did you answer them?

B: It doesn't seem to be, because I haven't played myself.

A: Will I be willing to tell others in the future, that is to say, let others get HPV vaccine, or to popularize the similar situation of HPV vaccine?

B: If I have no reaction after playing, I will certainly popularize science. As long as you fight and you have not played, people may come to ask you, then I will truthfully say that this thing is still ok, but I don't know the preventive effect. I can only popularize science with others this thing without any big adverse reaction.
