# Supplementary material for: Understanding cultural perceptions of sexuality in China and their influence on human papillomavirus vaccine hesitancy
Source: Front Public Health. 2025 Jan 23;12:1462722. doi: 10.3389/fpubh.2024.1462722 (PMC11801254; doi:10.3389/fpubh.2024.1462722)
Supplement: Supplementary file 1 [file Data_Sheet_1.zip › Frontiers_Supplementary_Material/Interview Transcripts - Participant 10.docx]

**Interview Transcripts - Participant 10**

A: Let's start by discussing your understanding of the HPV vaccine. Where did you first hear about HPV?

B: The first time, I think it was after my college entrance exam. My mom and dad were discussing whether I should get vaccinated. Yeah, my mom mentioned that she saw many people getting vaccinated and suggested I should consider it too.

A: That's interesting because many people I've interviewed learned about HPV vaccines primarily online. How did you come across information related to HPV infections and the vaccine?

B: From what I know, it can prevent certain diseases. I've looked up information online about the vaccine's effectiveness in preventing cancer. However, the results seemed to indicate it only prevents certain types, not all. I also learned there are different types like the 4-valent and 9-valent, but I don't have a deep understanding because I haven't actually gone to get vaccinated. I think I would delve deeper into understanding the vaccine if I made an appointment to get vaccinated.

A: Have you come across any information regarding how HPV infections occur, or its transmission pathways?

B: I have seen some information, but I don't remember it clearly. It seems people often talk about it in relation to sexual behavior, suggesting it's a serious disease, almost like a sexually transmitted disease. However, I've also read that it can be transmitted through other means besides sexual contact.

A: Right, we can delve deeper into a topic I plan to discuss later. Many people find it uncomfortable to discuss sexual topics in daily life, including how HPV infections occur. Do you think this is due to societal taboos?

B: I think so, because when people talk about vaccines like the rabies vaccine, they do so openly. Sometimes they even post on social media about getting vaccinated. But when it comes to HPV vaccines, people may mention they got vaccinated but not necessarily discuss its preventive aspects. If someone actually gets infected, they probably wouldn't mention it anymore.

A: Exactly. Since HPV infections are often associated with sexual behavior, there's reluctance to discuss cultural issues like premarital sex. You mentioned earlier that some people view it as a sexually transmitted disease. Do you think HPV symbolizes moral stigma, like punishment for promiscuity, or that getting vaccinated might imply being viewed as impure?

B: Yes, I think these perceptions exist in society, otherwise why would so few people discuss it openly? Personally, I don't see it as a symbol of impurity. Even if it's transmitted through sexual activity, similar to diseases like HIV or other STDs, I wouldn't judge someone as morally inferior. I'd think they're unfortunate to have contracted it. Blaming someone's personal life choices for their illness doesn't seem right to me, as there's not necessarily a direct correlation between personal life and illness.

A: Right. Do people around you, particularly older generations, hold such views? You mentioned some view HPV as a sexually transmitted disease. Is this more from what you've seen online or from personal interactions?

B: I think I encounter these views more in personal interactions. I don't discuss these topics with older generations much, but with peers during dormitory discussions, we sometimes delve deeper into such topics. Most of us share a similar attitude of questioning why such topics are considered taboo or why people avoid discussing sexual issues.

A: So, when you mentioned earlier about the stigma surrounding sexual matters, you view it critically, right?

B: Yes, I think it's unnecessary.

A: Would you actively take measures to prevent HPV infection?

B: Personally, I haven't thought much about it because I understand it's mainly transmitted through sexual relations or other means. I feel I lead a clean lifestyle, so I don't think I would get this disease. Some people might contract it due to poor habits, so if I were to make an effort, I might focus more on personal hygiene.

A: Based on your current understanding of HPV vaccine information, how inclined are you to get vaccinated? If you had to rate your willingness on a scale of one to ten, where would you place yourself?

B: I'd rate my hesitation at a five, right in the middle.

A: What are the main reasons for your hesitation?

B: My hesitation stems from what I've seen online. Some people share experiences of experiencing side effects after vaccination. Given my poor health condition, I worry I might experience similar side effects if I were to get vaccinated. Another concern is that despite getting vaccinated, some still contract the disease. Knowing that the vaccine only prevents certain types makes me question its effectiveness. Also, it seems quite difficult to schedule an appointment nowadays, so I haven't pursued it.

A: You mentioned observing certain side effects online. Could you specify what side effects you've seen?

B: Some have irregular menstruation or skin issues. Symptoms vary among individuals, but they all occurred after vaccination. Some experienced minor illnesses like headaches or feeling weakened.

A: I've come across similar posts online myself. When it comes to taking medications, are you similarly cautious about potential side effects, or is this specific to vaccines?

B: Honestly, I research potential side effects of medications or anything I ingest to be prepared in case I experience any. However, I'm more cautious about vaccines because their potential side effects seem more severe and diverse.

A: Overall, would you describe yourself as someone who tends to be conservative in avoiding vaccine risks, or are you more open to trying new things?

B: At the moment, I lean more towards being conservative. I believe in the continual advancement of medical technology, hoping better vaccines with fewer side effects will be developed before I consider getting vaccinated.

A: So, it's more of a wait-and-see approach. Besides what you've mentioned—online side effects and doubts about the vaccine's universality or scheduling issues—are there any other personal factors influencing your decision? For instance, mistrust towards healthcare providers or the healthcare system?

B: I actually trust the medical field. Even if they aim to profit, I don't think they'd recommend something harmful to my health. My concerns mainly revolve around the vaccine itself. Also, I've noticed few people my age or peers have gotten vaccinated, at least from what I've seen.

A: Could this be influenced by a herd mentality? If more people were vaccinated, would you perceive it as more necessary and perhaps follow suit?

B: If many people did get vaccinated, I might start considering it more necessary and follow suit. But since I haven't seen many people around me get vaccinated, I'm not particularly inclined.

A: That's an interesting point. Have you noticed a period when there was a significant media push for vaccination? Perhaps around 2021 or 2022?

B: Yes, during that time, after I finished my college entrance exams, I started paying more attention to internet discussions. I remember several classmates getting the 9-valent vaccine that summer. The latest I recall was around my sophomore or junior year, but over the past year, I haven't seen many people actively discussing or getting vaccinated. It seems after the initial rush, posts about vaccine side effects began to surface online again.

A: So, the proportion of people around you who have actually gotten vaccinated seems quite low, and it was just a specific period when many went for it. You mentioned rating your willingness to get vaccinated at 5 out of 10. If you were to consider getting vaccinated in the future, what might prompt you to do so? What would lead to a change in your mindset towards getting vaccinated?

B: If I were to get vaccinated, it might be because I realize the vaccine isn't as frightening as I initially thought. Or perhaps I'd ask my vaccinated friends about their experiences to see if what's said online about side effects is biased due to survivorship bias. Even though many report side effects online, I wonder if those who don't experience any effects simply don't speak up. My rating of 5 doesn't mean I'm completely against it right now, but I also don't feel a pressing need to get vaccinated immediately. Plus, there's an age limit, so I think I can wait until I need it, like when I become sexually active. At that point, I might inquire more and make a decision after understanding more about the side effects.

A: Alright. Let's talk about the sources of information. Do you think you've gathered more information about the HPV vaccine online or offline?

B: When it comes to supplementary information like others' experiences with the vaccine, that's mainly online. However, for practical details such as making appointments, which type of vaccine is suitable for me, and pricing, that information mainly comes from offline sources. My parents handle these things and offer recommendations.

A: So discussions at home about vaccines are quite frequent, you mentioned?

B: We have brief discussions, like at the dinner table we might discuss whether or not to get vaccinated, the fact that many people have been vaccinated, concerns about side effects, and whether it's necessary to get vaccinated now or if appointments are available.

A: But you haven't reached a decision yet on whether or not to get vaccinated?

B: Exactly. Currently, my family is adopting a wait-and-see approach. We want to gather more information before making a decision.

A: When discussing with your parents, since vaccines are related to sexual activity, do you find it awkward to discuss, or is it a topic that's open for discussion?

B: Honestly, we've never discussed the specifics of how vaccines relate to sexual activity. Our discussions have been more about how the vaccine works, what it prevents, and how one might contract the disease. I've had to find out about these things on my own. Initially, I thought it was just a regular vaccine.

A: So, do you think they understand how the vaccine works, or is it more of an unspoken understanding where no one really discusses the details?

B: I think they should know, because it's meant to prevent cervical cancer, right?

A: Exactly. It's meant to prevent cervical cancer after infection.

B: Because I remember seeing a lot of promotional materials about gynecological diseases when I was young. There were many brochures and small books distributed by specialized hospitals in our area. Even back when I was in elementary school, they promoted treatments for cervical cancer and cervical erosion. So, I've known about these terms since I was young. Now that I've looked into it further, I understand it's related to sexual activity.

A: Got it.

A: Besides discussions at home, have you also talked about this with friends and classmates?

B: Yes, I haven't discussed it much with classmates because I don't have strong intentions of getting vaccinated myself right now. So, I haven't talked to them about it yet. When we do talk, it's usually about topics related to sex.

A: Right, it seems that the conversation sometimes shifts to the topic of vaccines causing some to think about getting infected with HPV or even cervical cancer, which is a sexually transmitted disease.

B: Personally, since I knew about these terms since I was young, I don't get startled like I might if someone mentioned syphilis as a sexually transmitted disease. I don't really connect it to sex; I just thought it was a regular disease. It was only after I learned more that I realized it could be transmitted through sexual contact.

A: Your situation seems unique. In previous interviews, many mentioned avoiding discussions about sex-related topics. Roommates might only discuss how to get vaccinated and make appointments, skipping the reservations altogether.

B: Yes, since neither my roommates nor I have been vaccinated, we haven't discussed whether to get vaccinated. After the initial hype died down, it's not something we talk about much. Maybe our parents would ask if we want to get a vaccine to prevent future diseases. Among friends, we sometimes discuss topics related to our majors. In our field, such topics might not come up much. Our professors do occasionally discuss various aspects of sex and gender in class, so it doesn't feel awkward during casual conversations.
