# Supplementary material for: Understanding cultural perceptions of sexuality in China and their influence on human papillomavirus vaccine hesitancy
Source: Front Public Health. 2025 Jan 23;12:1462722. doi: 10.3389/fpubh.2024.1462722 (PMC11801254; doi:10.3389/fpubh.2024.1462722)
Supplement: Supplementary file 1 [file Data_Sheet_1.zip › Frontiers_Supplementary_Material/Interview Transcripts - Participant 11.docx]

**Interview Transcripts - Participant 11**

A: Regarding the virus, could you give a general overview of what you understand about it?

B: HPV, from what I've seen in reports, is related to things like genital warts caused by the virus, right? I remember there are recommendations to get vaccinated, ideally before the age of 26, for better effectiveness. However, I haven't gotten vaccinated yet because my mom thinks it might not be reliable to get vaccinated in China. She prefers to take me to Hong Kong for the vaccination, believing it might be better there. I don't have all the details; she mentioned it to me before.

A: Okay, let's discuss this. Does your mother have a medical background or professional insights into vaccines, or is this assessment based on information she's heard from others?

B: She doesn't have a professional medical background. She probably heard from acquaintances and discussed it, mentioning that vaccines in China might not be very reliable. She thinks vaccines like HPV might have been available in Hong Kong first and might be more mature there. People who work there have already been getting vaccinated for some time, so she thinks it might be better than vaccines in China.

A: Okay, that's a common behavior. In previous interviews, many mentioned classmates traveling to Hong Kong or Macau specifically to get vaccinated.

B: Yes.

A: Moving on to the virus itself, what's your perception of the prevalence of HPV? For example, do you know anyone who has been infected or have you seen posts online from people claiming to have been infected? Your personal observations would be valuable, not necessarily statistically significant.

B: Currently, maybe because I'm still in school and not yet at the stage of marriage, it seems like none of my friends have mentioned being infected. I might see some posts online about HPV infections, and bloggers might give advice on precautions in sexual or other contexts to avoid contracting the virus. I might have heard from my mom or other older relatives that they know many women who have common gynecological diseases, though not necessarily HPV, which seems quite common.

A: Okay, earlier you mentioned seeing more advice online about how to protect oneself from infection rather than people sharing that they've been infected. Got it. Moving on to some knowledge about the transmission routes of HPV, do you know how HPV is transmitted? For example, like HIV, its transmission routes may include 123.

B: It's sexually transmitted.

A: Have you heard about other ways of transmission?

B: Apart from sexual transmission, there's also the possibility of transmission in places like hotels, where disposable items should be used, such as when using the restroom, to prevent infection. There's also the possibility of transmission through urine.

A: Do you know what specific diseases this virus might lead to? You mentioned some earlier.

B: Immune suppression and physical discomfort might occur because it's a gynecological disease. I haven't really looked into specific diseases caused by it. It's often compared to HIV in terms of severity. It could be treatable in the early stages but might become more difficult to treat in the later stages. Is that correct? Okay, thank you.

A: Earlier, we mainly discussed your understanding of the virus itself. Now, let's talk about vaccines. Could you share what you know or understand about vaccines?

B: Okay. Regarding vaccines, I've seen information on platforms like public accounts and news reports. For example, there were announcements about HPV vaccines being available at some health centers in our county under Shaoxing, Zhejiang. When they first came out, however, it was difficult to make an appointment or get the vaccine. At that time, I wasn't sure if I wanted to get vaccinated or not. I had this feeling that getting vaccinated was like admitting I might get the disease, almost like cursing myself. Maybe because I couldn't secure an appointment, I felt a bit resigned. Also, since I don't think I'm at the age to get married yet, I didn't think I would contract the disease. But now, I think it's necessary to get vaccinated. My perspective has changed because now it seems easier to make appointments. During winter break, I tried twice to schedule an appointment, though I didn't succeed. I still feel there's a chance, though, because many people are getting vaccinated, so there's still an opportunity to schedule an appointment and get vaccinated. I believe it would be beneficial for my own preventive care.

A: So, you mentioned you might get vaccinated before you turn 26, but you haven’t done it yet. Is it because of scheduling issues, or are there other concerns?

B: I plan to get vaccinated before I turn 26, but I haven't been able to make an appointment yet. There are some concerns as well, mainly from my mom, who thinks vaccines in China might not be very reliable and prefers to take me abroad for it. So, it's still under consideration. But I do plan to get vaccinated before 26.

A: What do you know about the types and costs of the vaccines?

B: It seems to be around 1,000 yuan per dose, and since it requires three doses, it totals about 4,000 yuan. This is a bit of a concern for me because I'm still in school. I feel like I should be earning my own money rather than asking my parents for it. I think once I start working, I’ll be more willing to pay for the HPV vaccine myself.

A: Do you know the different types of HPV vaccines available?

B: Yes, there's the 9-valent, 2-valent, and the 4-valent vaccines.

A: Do you know the age range for vaccination for these types? You mentioned it briefly earlier.

B: The 9-valent is for people under 26, and the others can be given up to 30 or 50 years old, I believe. I remember reading that somewhere.

A: Alright. Do you know how long the protection from the vaccine lasts?

B: I'm not really sure. I think it might be lifelong, but I haven't seen specific information on that.

A: That's fine. Lastly, regarding the vaccine itself, who do you think would most benefit from getting the HPV vaccine?

B: I think all women, especially those who are becoming adults, should consider getting it. Basically, any woman who wants to prevent HPV-related diseases should get vaccinated if they are aware of it.

A: Earlier we talked about the virus and vaccine-related knowledge. Based on what you know, where do you think most of your information about the vaccine comes from? Online sources, friends, or family?

B: I think most of it comes from online sources, like public accounts on social media, health center public accounts, and short videos on platforms like TikTok. I follow a TikTok account that focuses on women's health and prevention, which often shares knowledge about HPV prevention. Also, some information comes from family, especially older relatives who give advice on women's health. Friends don’t really talk about it much unless it’s about whether they’ve gotten the 9-valent vaccine or not.

A: Why do you think people don’t talk much about this topic offline? From your perspective, why might there be reluctance to discuss vaccine information in person?

B: I think it might be due to a lack of awareness or knowledge about women's health and related issues. It’s not necessarily avoidance, but there’s still some psychological barrier, similar to the stigma around menstruation. People are not very comfortable discussing women's health issues openly, like cervical cancer or other gynecological conditions. There’s a certain level of discomfort or shame that prevents these discussions.

A: Are you referring to cervical cancer or other gynecological conditions?

B: Yes, cervical cancer and other women's health issues. People don't usually talk about these things openly. There's a lack of widespread information and awareness about these topics. However, with the introduction of the 9-valent vaccine, there's been more focus on women's health and gynecological diseases. Before that, I think people were less aware and didn’t pay much attention to these issues. Online information wasn’t as prevalent, and there was a sense of shame about discussing these topics in person.

A: So, during your process of understanding the vaccine and the virus, did you encounter any obstacles in obtaining accurate information? For example, finding out the suitable age for vaccination, coming across contradictory information, or even conspiracy theories? Have you experienced such difficulties?

B: Yes, definitely. Initially, I heard that people up to 35 could get vaccinated, then it changed to 26. I encountered a lot of incorrect reports, which were later corrected. This led to confusion. I think the dissemination of accurate information by health authorities is still lacking, causing public misinformation.

A: Last question for this section. Based on all the information we've discussed, how would you rate your understanding of the virus and the vaccine? Do you feel you have a basic understanding, a comprehensive understanding, or do you think you still lack sufficient knowledge?

B: I think I still don’t fully understand it. I know that the vaccine can prevent cervical cancer, but I haven't delved deeply into the specifics of the disease itself.

A: Alright. Let's move on to the third part about vaccine hesitancy. Earlier, you mentioned some hesitancy, such as waiting until you’re closer to 26. Now, let’s discuss your specific concerns about the HPV vaccine. You can list them, such as financial reasons, etc.

B: First, the financial aspect is a concern since I'm still in graduate school and don't have a lot of savings to spend on the vaccine. Second, as I mentioned earlier, my mom is worried about the efficacy of domestic vaccines compared to those in Hong Kong or Macau, so we haven't decided which one to get. Third, scheduling is an issue. I’m studying in another city, so it’s hard to go back and forth for the three doses required. It’s inconvenient to return for each shot, considering the time intervals between doses.

A: I see. We’ve already touched on some aspects of this, like your concerns about age suitability for the 9-valent vaccine and the timing.

B: Yes, I remember reading that the vaccine should ideally be given before 26. When the vaccine first came out, the age range was quite broad, suggesting people in their 30s or 40s could get it. But then I read on a public account that it’s most effective before 26, and they strongly recommend getting it by then. Since I’m now 24, I’m keeping an eye on my age and planning to get it within the next two years.

A: Could you share your thoughts on the stigmatization of the HPV vaccine? Please explain what stigmatization means in this context, including any stereotypes associated with the HPV vaccine. For example, some older generations might associate the vaccine with negative connotations about sexual impurity. You can briefly discuss your views on this stigmatization and these stereotypes.

B: I once saw a short video where a girl around my age went to get the vaccine with her mother. The doctor asked her, in front of her mother, if she had ever been sexually active. It was a very awkward situation. Watching that video made me worry that the same thing might happen to me. Why would getting a shot involve such personal questions?

A: Right.

B: The 9-valent vaccine is indeed related to sexual activity, but I think the anxiety around it is exaggerated by platforms like TikTok. When I asked my friends who had gotten the vaccine if they were asked about their sexual history, they said no. The process was straightforward, and they didn't encounter any awkward questions. Even if doctors do ask about sexual history, it's part of their routine inquiry. The awkwardness arises because some older people equate gynecological diseases with sexual impurity and shame. They fail to see these issues as separate. Many people, when discussing HPV, immediately associate it with sex and, consequently, with the notion of female impurity. This stigmatization is unfair and unjust to women.

We need to promote correct knowledge about gynecological health and encourage open discussions about the HPV vaccine. It's important for these conversations to take place openly, even at family gatherings, where older generations can share their knowledge about gynecological health without shame. This is my understanding of the situation.

A: Great, that's a perfect way to wrap up our interview. We’re done here.
