# Supplementary material for: Understanding cultural perceptions of sexuality in China and their influence on human papillomavirus vaccine hesitancy
Source: Front Public Health. 2025 Jan 23;12:1462722. doi: 10.3389/fpubh.2024.1462722 (PMC11801254; doi:10.3389/fpubh.2024.1462722)
Supplement: Supplementary file 1 [file Data_Sheet_1.zip › Frontiers_Supplementary_Material/Interview Transcripts - Participant 12.docx]

**Interview Transcripts - Participant 12**

A: Can you first talk about what you know about this virus?

B: I remember the virus is supposed to prevent several diseases in women. I can only name cervical cancer specifically. Then there are different types like the 9-valent, 2-valent, and 4-valent vaccines, with some being imported and others not. It seems the 9-valent covers the most types.

A: And if you had to mention one thing, you would say cervical cancer?

B: Yes, that's all I can recall.

A: Do you know anything about the prevalence of this virus? Have you heard of anyone around you being infected or seen information about it online?

B: No one I know has it. Does breast cancer count? It doesn’t seem related to HPV. I haven't seen much about it online either. When I was younger, in elementary or middle school, I remember seeing pamphlets from hospitals boasting about their gynecology departments and mentioning that cervical cancer and cervical erosion could be treated. But beyond that, I haven't come across much information.

A: So, based on what you know, do you think the virus isn't very prevalent since you haven't heard of many people being infected?

B: I think it might be because the people around me have stable partners, so it doesn't seem like a big issue. But I believe the virus is probably quite common; I just haven't encountered anyone in that group, similar to how HIV is prevalent but not something I come across personally.

A: Understood. Next, do you know the transmission routes of this virus?

B: The main route I can think of is sexual activity. If people don’t have a stable partner, the risk of infection is higher. With a stable partner, the risk is lower, but if the partner is unfaithful, the risk increases. Lack of protection and existing diseases in a partner also play a role. I think personal hygiene might contribute, but not significantly.

A: And if someone is infected with this virus, do you know what diseases it can cause? Are these diseases severe or just mild symptoms?

B: Infection can range from mild to severe. Mild symptoms might include inflammation or abnormalities. Severe cases can lead to diseases like cancer, especially if the partner is also infected.

A: Moving on from the virus, let's talk about the vaccine. You’ve already mentioned some points. Can you elaborate on your understanding of the vaccine?

B: Sure, as I said, there are different types like the 9-valent, 4-valent, and 2-valent vaccines, with both domestic and imported versions. The 9-valent covers the most types and is hard to book. I know friends who’ve gotten it, and booking the 9-valent took a long time. When I went with a friend for their vaccine, the doctor suggested that the 2-valent or 4-valent is sufficient for people with stable partners, as it covers enough types. So, I think if I get vaccinated, I would go for the 4-valent or 2-valent. My cousin in middle school is getting vaccinated, and their school is offering the 2-valent or 4-valent. The 9-valent is hard to get, and I don’t think I’ll need it since I plan to have a stable partner.

A: You mentioned the doctor said the 2-valent or 4-valent is enough. Can you explain more about what the doctor said during your visit?

B: The doctor confirmed our details and mentioned that there’s no need to follow the trend of getting the 9-valent vaccine, as it’s in high demand and expensive. The doctor, who was in their forties like my mom, said that the 2-valent or 4-valent is sufficient for most people.

A: Yes, the prices from scalpers can be very high. Who do you think needs the HPV vaccine the most, particularly the 9-valent one?

B: I feel that the vaccine is best administered before engaging in sexual activity. For personal health reasons, I think it depends on individual needs. Ideally, I'd like to get the 2-valent vaccine with my mom because she's at an age where only the 2-valent vaccine is recommended. I'm concerned that as she ages, she might be at risk.

A: You mentioned age earlier. Do you know the age ranges for the 2-valent, 4-valent, and 9-valent vaccines?

B: I know they differ, but I haven't paid close attention to the exact ranges. I think the 9-valent vaccine can be given starting at age 9, right?

A: Yes, from age 9.

B: Right. And the 4-valent vaccine, I think, covers a broader age range than the 9-valent. But I'm not too sure about the specifics.

A: That's fine. Based on the information you've gathered about the virus and the vaccine, how well do you think you understand them?

B: A couple of years ago, when the topic was trending, I would see posts about people getting the 9-valent vaccine on platforms like Douyin (TikTok) and Xiaohongshu (Little Red Book). Initially, I confused HPV with HIV. But after some research and talking with friends who got vaccinated, I learned that it's mainly to prevent certain diseases in women. My knowledge is limited to knowing that getting vaccinated could potentially reduce the risk of some illnesses. I remember reading that the vaccine has a validity period and doesn't provide lifelong immunity. Also, I saw a post on Douyin saying that the 9-valent vaccine is a scam, claiming it wouldn't protect one's long-term sexual health.

A So, your understanding is somewhat limited, mainly focusing on specific aspects rather than comprehensive knowledge. You've mentioned gathering information from various sources. Which channels do you rely on most for information about the virus and the vaccine? Family, friends, or online sources?

B: I first heard about the vaccine on Xiaohongshu. I wanted to understand what it was, so I did some research and shared the information with my friends, as they were also unaware. I even talked to my mom about it, and she supported the idea of getting vaccinated. So, Xiaohongshu was a significant source of information for me.

A: You mentioned sharing information with your friends. How did those conversations go?

B: During classes or casual chats with friends and roommates, I brought up the HPV vaccine, mentioning it could prevent certain diseases. I asked if they wanted to get vaccinated together. I'm quite health-conscious and worry about getting sick. One friend declined because she felt her parents wouldn't understand or support it. Another friend, who was in a relationship at the time, did her research and eventually booked an appointment for the 9-valent vaccine, though it took over six months to get it. Another friend got the 2-valent vaccine, and my cousin got either the 4-valent or 9-valent, I can't remember exactly. They all reported positive experiences without any side effects. However, later on, I saw many online posts claiming that the 9-valent vaccine had numerous side effects and that traditional Chinese medicine practitioners advised against it, which confused me.

A: Considering all the information you've seen about the vaccine, including educational content and reports of side effects, do you find this information helpful? Or does it create confusion for you, seeing both the educational content and the side effects?

B: Yes, it does create some confusion. Initially, I was quite eager to get vaccinated because some of my friends experienced some irregularities with their periods after getting the vaccine, but it normalized the following month. Before seeing any side effect reports, I was keen on getting it, but since I didn't manage to book an appointment, I let it go. After reading about the side effects, I felt relieved I didn't go through with it. Even though I’m unsure about the accuracy of these side effects, such reports make me anxious. As a non-professional, I find it hard to discern which information to trust. Negative information especially makes me hesitant.

A: Alright, let's move on to the third part of our discussion about vaccine hesitancy. On a scale of 1 to 10, how would you rate your level of hesitancy?

B: Is this a scoring system? Sure, if 10 is the maximum, I would rate my hesitancy around 6.5 to 7.

A: You mentioned feeling indifferent or not particularly compelled to get the vaccine. Could you list your reasons or concerns in detail?

B: One reason is the side effects. Another is my laid-back attitude—if I don't manage to do something when I first intend to, I often don't pursue it further. Additionally, I don't foresee being sexually active with multiple partners anytime soon, so I don't see the urgent need. Also, I sometimes have irregular periods and prefer using traditional Chinese medicine for treatment, which I trust more.

A: So, you don't really trust or prefer Western medical methods, like vaccines, to protect your health?

B: Another issue is the vaccine's limited duration of effectiveness. If a vaccine doesn't provide complete, lifelong protection, I question its necessity.

A: We've discussed many of your thoughts and concerns. Let's start with your concern about side effects. You've heard about these from friends and online. After learning about these side effects, did you research whether these claims were true or isolated cases?

B: Yes, I did some research online. The more I searched, the more hesitant I became. I remember reading about someone who never experienced menstrual cramps before getting the 9-valent vaccine but started having them afterward. As someone who already suffers from cramps, that was alarming to me. Online searches often lead to more content about side effects due to data algorithms, reinforcing my concerns.

A: Have you ever sought information from official medical sources or healthcare providers to verify these claims or to find myth-busting articles about the side effects?

B: No, I haven't. I mainly use platforms like Baidu and Xiaohongshu, where personal experiences are shared, but there aren't many official accounts providing detailed explanations or debunking myths.

A: Let’s discuss the balance between the risks and benefits of the vaccine. You know it might have some side effects, but it also offers protection. How do you personally weigh these risks and benefits?

B: I think it depends on the timing. If I have a boyfriend in the future, I would get vaccinated because I’d be worried about his cleanliness and my health. I take my health seriously, but I can't guarantee my partner's health. I fear he might pass on some disease to me, so I see the vaccine as a necessary protection. The side effects become less of a concern if I'm at risk of contracting the virus, especially if I’m in a relationship and sexually active. In that case, I would consider the vaccine's benefits to outweigh the risks.

A: So, you believe the benefits outweigh the risks?

B: Yes, but currently, I don't feel an urgent need to get vaccinated because there's no immediate reason driving me to do so. If I were in a relationship, I’d consider it because the benefits would then clearly outweigh the risks to protect myself.

A: Earlier, we talked about the importance of getting vaccinated before becoming sexually active, right?

B: Yes, I know that. So, I would get vaccinated before being intimate with someone. It’s not like we plan to be intimate tomorrow and I get vaccinated today. My point is, once our relationship progresses to a certain level, I would preemptively get vaccinated.

A: You know the vaccine requires three doses over a specific period, right? You accompanied your friend, so you should be aware of this.

B: Yes, I know it requires three doses. I thought the first dose would offer some protection, maybe around 30%, and the subsequent doses would increase the protection level. I didn’t realize the protection takes that long to be fully effective.

A: Yes, the full protection comes after completing the three doses over a span of several months. The first dose is followed by the second dose a month later, and the third dose six months after the first.

B: I see, so the process takes longer than I thought.

A: How do you view the stereotypes or stigmatization surrounding vaccines and viruses? For example, some might think getting vaccinated implies promiscuity.

B: I think such opinions likely come from men. Vaccines are a protective measure, like the many vaccines we receive as children. It’s absurd to associate vaccination with promiscuity. Women get vaccinated out of self-love and concern for their health. Some people might spread diseases intentionally out of spite, as seen with certain individuals with HIV. Additionally, outdated or conservative mindsets, especially from older generations, might lead to such stigmatization. It’s a matter of evolving perspectives.
