# Supplementary material for: Understanding cultural perceptions of sexuality in China and their influence on human papillomavirus vaccine hesitancy
Source: Front Public Health. 2025 Jan 23;12:1462722. doi: 10.3389/fpubh.2024.1462722 (PMC11801254; doi:10.3389/fpubh.2024.1462722)
Supplement: Supplementary file 1 [file Data_Sheet_1.zip › Frontiers_Supplementary_Material/Interview Transcripts - Participant 13.docx]

**Interview Transcripts - Participant 13**

A: To start, could you share your understanding of this virus?

B: From what I know, this virus is related to cervical health. It comes in various types, categorized into low-risk, medium-risk, and high-risk. High-risk types have a higher chance of causing cancer, while low-risk types are associated with other gynecological issues.

A: How prevalent do you think this virus is? Have you seen or heard about people getting infected, either around you or online?

B: I’ve seen a lot of discussions online, particularly in a Douban group. Many women have reported infections of varying risk levels. My mother was diagnosed with a low-risk type at the end of last year, specifically types 34 and 39, among others. While most of my friends are single and I haven’t heard of any infections directly, I believe the virus is quite common.

A: You mentioned joining a Douban group. Is it focused on women’s health or specifically on HPV?

B: It's more about women’s health in general, not specifically HPV.

A: So, people in the group share knowledge related to women's health?

B: Yes, that's correct.

A: What made you decide to join a women’s health group to learn more about this topic?

B: About two years ago, there was a lot of talk about HPV vaccinations among people around me. I thought about getting vaccinated and wanted to learn more, so I joined the group.

A: I see.

B: Also, in another group I’m part of for following celebrities, someone posted about gynecological issues, which led me to learn more about the topic.

A: Understood. How do you think this virus is transmitted?

B: Mainly through sexual contact. Other methods, like contact in hot springs, are possible but less common. I’d say 97% of cases are sexually transmitted.

A: You mentioned earlier the diseases this virus can cause. Do you think these diseases are usually severe, or are most cases relatively mild?

B: It depends on the type of infection. High-risk types can be quite serious.

A: Do you think people are more likely to get infected with low-risk types or high-risk types?

B: I think low-risk infections might not always be detected because they cause milder symptoms that can be treated with other medications. High-risk infections are more likely to be discovered because they are more severe.

A: Now that we’ve discussed the virus itself, let’s focus on the vaccine. Could you share your understanding of the vaccine?

B: The vaccine comes in three types: 2-valent, 4-valent, and 9-valent, covering different strains of the virus. There are also domestic and imported versions, with the latter generally believed to be more effective.

A: Does the difference between domestic and imported vaccines significantly influence your decision to get vaccinated? For instance, would you prefer getting vaccinated in Macau or Hong Kong over using a domestic vaccine?

B: I’m not too concerned about that. For me, the cost is more important.

A: Who do you think is most suitable for vaccination?

B: There’s an age limit. The 9-valent vaccine is now available from age 10 to 45.

A: Yes, that’s correct.

B: The 4-valent vaccine used to have a broader age range, but now the 9-valent has also been expanded. I remember the optimal age range for vaccination is up to 26 years old.

A: So, there are two things you know: the general age range and the optimal age range being up to 26 years.

B: Yes, and I also know that men can get vaccinated, though it's mostly women who do.

A: Based on the information you've shared about the virus and the vaccine, how would you rate your understanding? Do you feel well-informed, or just have a basic knowledge?

B: I’d say I have a moderate understanding. I haven't gone into deep specifics, but I know enough to make an informed decision about getting vaccinated.

A: You’ve mentioned getting information from various sources. Could you elaborate on where you primarily get this information? For example, do you mostly find it online or through community health centers?

B: My main sources are the internet and conversations with friends and family. Online, the primary platforms are Xiaohongshu, Douban, and Weibo. As for personal interactions, it's mostly discussions with friends of my age and some talks with my mom.

A: Let's start with offline sources. You mentioned that only your mom has discussed this topic with you. Do your classmates or friends not talk about it?

B: My mom and my friends do talk about it. My friends and I discuss it quite frequently.

A: What do you mainly talk about regarding the vaccine? Is it more about how to book an appointment, your experiences post-vaccination, or the risks of infection?

B: We mainly discuss whether to get vaccinated and share tips on booking appointments, like the best times and places to secure a spot. We also talk about our experiences post-vaccination but rarely discuss the virus itself or the risk of infection.

A: So, you focus on the logistics of getting vaccinated and the experiences afterward, rather than detailed discussions about the virus and its transmission?

B: Yes, that's correct. We rarely discuss the virus in detail.

A: Why do you think you and your friends focus more on the logistics and post-vaccination experiences rather than the virus itself?

B: My friends mostly associate the virus with cervical cancer and feel strongly about getting vaccinated to prevent it. They are more concerned with the practical aspects of vaccination rather than the detailed science behind the virus.

A: Understood. When you talk to your mom, what do you discuss? Is it similar topics, or do you go into more detail?

B: Initially, my mom would ask if I wanted to get vaccinated and where I preferred to get it done. As I learned more, our discussions expanded to include details about the virus and ways of transmission.

A: So, you and your parents are comfortable discussing topics like transmission methods, including sexual transmission?

B: Yes, we are open about those topics.

A: That covers offline sources. Moving to online sources, you mentioned finding a lot of information on the internet. What kind of content do you usually come across? Is it more about vaccine types, personal experiences, or general information about the virus?

B: The content I find mainly falls into two categories: personal vaccination experiences and discussions on whether getting vaccinated is necessary.

A: So, the information you encounter is primarily about vaccination experiences and the decision-making process, rather than in-depth details about the virus itself?

B: Yes, that’s right. The information I see aligns with what I already know, focusing on similar aspects rather than providing deeper insights.

A: Do platforms like Xiaohongshu actively push this information to you, or do you search for it yourself?

B: I actively search for this information myself.

A: So, you proactively seek out information about the virus and the vaccine?

B: Yes, because Xiaohongshu’s algorithm doesn’t prioritize this kind of content for me unless I look for it specifically.

A: If there’s conflicting information between online and offline sources, which one do you tend to trust more? For example, if many people online say they experienced pain or side effects after vaccination, but your friends in real life say they had no side effects, which would you believe?

B: I don’t really take that kind of information into account. I think it’s a matter of individual differences, so it doesn’t influence my personal decision. It’s not about believing one over the other.

A: So, do you have a preference between online and offline information?

B: I might lean more towards offline information.

A: So, you trust what you hear or see in person more, though you recognize there are individual differences?

B: Yes.

A: Moving on to vaccine hesitancy, can you describe your current level of hesitancy about getting vaccinated? You can give it a score out of 10 if you like.

B: I’m not hesitating. I’ve decided not to get vaccinated, at least not this year or next year. I don’t see the necessity, considering the transmission routes don’t apply to me. Plus, the cost is quite high.

A: I see. Have you tried to book an appointment and found it too troublesome, with things like lottery draws or long waits?

B: Yes, I have tried.

A: Regarding transmission routes, besides sexual transmission, there are other ways HPV can spread. Are you worried about those other routes?

B: I am a bit concerned, especially about places like public hot springs. However, I don’t frequent such places often, and my living environment is relatively stable and closed, so I’m not overly worried.

A: So, there is some concern, but not enough to drive you to get vaccinated?

B: Right.

A: You mentioned the optimal age for vaccination is 26. Don’t you think it’s better to get vaccinated as early as possible for the best effectiveness?

B: The optimal age is ideal, but even if you’re past it, the vaccine is still somewhat effective. Also, getting vaccinated doesn’t guarantee 100% protection against infection.

A: So, the optimal age isn’t a strong enough reason for you to get vaccinated immediately?

B: No, it’s not.

A: Do you know that the HPV vaccine requires three doses?

B: Yes, I know.

A: And are you aware of the intervals between the doses?

B: Yes.

A: Do you know how long the intervals are?

B: One month and six months, right?

A: Right. So, don’t you think it’s best to get vaccinated before becoming sexually active, rather than after starting and then having to wait almost a year to complete the vaccination process?

B: I plan to get vaccinated before I become sexually active. If I’m not currently planning to date or have sex, there’s no need to get vaccinated now. If I decide to start a relationship and plan to have sex, then I’ll get vaccinated. If my partner can’t wait a year or respect my decision, then it’s not worth continuing the relationship.

A: Regarding the last point on pricing, how do you make value judgments? For example, you mentioned it costs around four to five thousand yuan. How do you perceive this cost—do you find it reasonable for things like travel but not for vaccines? What factors influence your judgment and motivation?

B: It’s not that I think four to five thousand yuan is particularly expensive; rather, I anticipate the price might decrease slightly in the future. If I'm not urgently in need of getting vaccinated right away, waiting a bit longer is fine. It's not about consumer perspective but more about expecting a potential price drop and being in no rush to get vaccinated.

A: I see. So, while you understand some benefits of the vaccine, there’s nothing currently motivating you to get vaccinated in the near future, such as starting a relationship and anticipating sexual activity prompting you to do so?

B: Exactly.

A: Alright. Moving on, the third part covers these aspects, and now onto the fourth part, which is optional. If interested, could you discuss your views on the stigmatization of HPV vaccines from a rational perspective? Could you briefly explain what stigma means in this context—how it might affect perceptions of the HPV vaccine? Do you see or have you encountered examples online or in your surroundings where people are troubled by such perceptions? How do you personally view these ideas?

B: In my circle—friends, family, and older generations—there isn’t much negative perception of the HPV vaccine. Everyone generally agrees it’s for better health and should be taken. As for stigma, it’s not just about the HPV vaccine but reflects a broader societal issue regarding female chastity in any topic. There’s a prevailing belief that blames women for things like being raped because of how they dress. Similarly, with vaccines, despite it being women predominantly getting vaccinated, the virus originates from men. It’s a superficial solution that doesn’t address the root cause; ideally, men should also get vaccinated, but some fear it might affect their masculinity.

B: The topic of sterilization also heavily impacts women’s health, yet men don’t face such consequences. Birth control pills are also predominantly taken by women. There’s an overarching societal exploitation and oppression of women’s sexuality in various contexts. However, I don’t encounter these views much in my personal circles.

A: So, these perceptions exist, although you haven’t observed them personally. Earlier, you mentioned these ideas stem from broader societal biases towards women, attributing blame to them for various issues, correct?

B: Exactly.

A: Alright, that wraps up my questions. Thank you for your insights across these four main parts of the interview.
