# Supplementary material for: Understanding cultural perceptions of sexuality in China and their influence on human papillomavirus vaccine hesitancy
Source: Front Public Health. 2025 Jan 23;12:1462722. doi: 10.3389/fpubh.2024.1462722 (PMC11801254; doi:10.3389/fpubh.2024.1462722)
Supplement: Supplementary file 1 [file Data_Sheet_1.zip › Frontiers_Supplementary_Material/Interview Transcripts - Participant 14.docx]

**Interview Transcripts - Participant 14**

A: Could you start by explaining what you know about this virus?

B: Sure. I'd like to ask about this virus. Yes, regarding this virus, I feel that primarily—firstly, I want to ask about proving whether one hasn't had sexual intercourse. Secondly, if one normally pays attention to personal hygiene, I feel the incidence rate of this virus can be reduced to below 10%. Previously, I asked a doctor because my personal inclination was not to get vaccinated. So, when I talked to that doctor, my mom asked me to go and ask the doctor, and then the doctor told me it's also fine not to get vaccinated. Because this is basically a minor issue, as the doctor told me, mainly because of sexual activity. Because males can carry the disease without symptoms, and the situation is different for females, they may show symptoms. So, as long as there is no sexual activity, the incidence of this disease can be reduced to less than 10%, which can prevent 90% of the virus. And the remaining part, about not paying attention to hygiene, there's still a small chance of contracting the disease.

A: Alright. How do you see the prevalence of this virus? Do you personally know anyone who has been infected, or have you seen many cases of infection online?

B: Yes, as you mentioned.

A: Are these people around you or people you've seen online?

B: Online.

A: Do they share their experiences of being infected online?

B: Yes, they do share. For instance, some people might share that they have been infected, and they would post on platforms like Xiaohongshu or Douyin, asking other users about when the disease might turn negative, and how to care for and heal from this situation.

A: So, overall, do you think it's widely prevalent, or is it more of a common virus, or do you believe that the number of infected people is actually quite small?

B: I feel that the number of infected people is quite small because the people I've been in contact with haven't been infected.

A: You mentioned the transmission routes earlier, right? And do you know what diseases might occur after someone is infected? Are these diseases severe, or are they more common, or perhaps not something to worry about at all?

B: I think when you get sick, you definitely need to be careful, but it's not like diseases such as HIV, hepatitis B, or syphilis, which are infectious diseases. It's just a disease that can be turned negative, so I think it's necessary to pay attention to it, but there's no need to excessively worry about it.

A: Okay. Moving on from the virus-related knowledge, let's focus on the vaccine, specifically the HPV vaccine. What do you understand about the vaccine?

B: It seems we're getting into the area I'm familiar with. Frankly, I'm not very resistant to receiving this vaccine. The reason is that our class also consulted a doctor at the time, and the conditions the doctor gave were, firstly, that it has an expiration date, because you know a vaccine is not cheap. If it's a single dose, it's also a few thousand yuan cheaper. However, it has an expiration date, it's also one year, if you want to guarantee it for decades, you may say to go and get vaccinated, it's just one year, and there's no need. Secondly, I saw online that some people would say there are various sequelae and so on. And some people around me have come into contact with vaccines. After the girl got vaccinated, her roommate would have a rash on her body and so on, and it was quite uncomfortable. So, I dare not get vaccinated. And the third reason is that it's still an expiration date after all, so you might have various situations later on, it might not be marriage or something, you always have sex, if you accidentally get infected, it's equivalent to getting vaccinated and it's useless, so I think so.

A: When you said the expiration date is one year, did you go and ask the doctor?

B: Yes.

A: Was it a doctor who got the vaccine at the community hospital, or was it someone from another department?

B: I don't know, my mom asked.

A: He asked about the situation, and the doctor said the expiration date is one year.

B: Yes, because I wasn't at home, he was at home asking, and he said he wanted me to go home and get vaccinated, so I said, why not ask the doctor first, and ask again.

A: You were doubtful about this at the time, let your mom ask, and then you said you didn't say to go online and say, I want to go and see what it is.

B: I've seen it, but this thing online, it's because people's physiques are different, and it will say that people's physiques may have sequelae, and some don't have sequelae, and some doctors will post saying that it's necessary, and some doctors will say that they don't think it's necessary, and online, how to say, everyone has their own opinions, and everyone says everything.

A: My thinking is that with negative news occupying a certain proportion, these messages aren't uncommon. I feel it's better not to take risks. It's not about believing anyone's words outright or thinking the vaccine itself is inherently bad. It's just that it varies depending on individual constitution, but I prefer not to take chances.

B: When I mentioned negative news earlier, I meant side effects and also the short shelf life of the vaccine.

A: You mentioned the side effects earlier, right? Are those the side effects you know about, or are there other negative aspects you're aware of?

B: Other side effects might lead to some physical discomfort.

A: Some people might say their menstrual cycle became irregular after getting vaccinated, or that their skin worsened.

B: Yes, and some people may experience other issues related to their personal constitution.

A: Did you check official accounts, like those from public health institutions, such as articles on Baidu Health, or did you seek advice from health professionals to see if there were any side effects?

B: Yes, I did.

A: Did you search for information?

B: My grandma mentioned that when I went to get vaccinated because I thought it related to my health. So, I collected information, and the more I collected, the more I found myself hesitating. Initially, when the vaccine came out, I was very eager and thought I must save up money to get vaccinated. However, as I gathered more information, I became more hesitant and didn't want to proceed.

A: So, it wasn't just from platforms like Xiaohongshu; you also searched on official sites. How did they explain it to you?

B: After searching on official platforms, some doctors would explain the benefits of getting vaccinated and whether not getting vaccinated would have a big impact.

A: Did these doctors run public accounts or was it somewhere else?

B: It was from online consultation.

A: Did you look for them and ask, and they told you the advantages and disadvantages?

B: Besides asking online doctors, I also asked local doctors before because I had irregular periods before, so my mom took me to see a doctor. After I added the doctor on WeChat, I asked her many things, including the vaccine. She presented objective facts, but the decision of whether to get vaccinated or not was still up to me.

A: That's good to hear. About the vaccine itself, you've mentioned a lot of information. How do you feel about your understanding of it? From what you've said, it seems you've done a lot of research. Do you feel you have a good grasp of it?

B: Yes, I've done a lot of research on various platforms and asked doctors.

A: Did you discuss vaccine-related topics with classmates or friends?

B: During that time, when the HPV vaccine was just released, we talked about it every day, especially since one girl's entire dorm got it. She asked me about it, and we've discussed it ever since. She mentioned her roommate and others, and that while five out of six people didn't have any issues, some experienced irregular periods, though she felt it wasn't a big deal. But there was one girl with poor health who had rashes all over her body, and she went to the hospital, so it seemed quite serious. I think what's said online might not seem like a big deal, and everyone has different constitutions, but seeing it offline with that girl with the rashes was quite scary.

A: Did you mainly discuss side effects, or did you talk about the difficulty in getting an appointment or other things?

B: It was hard to get an appointment at first, and my enthusiasm was strong at first, so I wanted to get vaccinated. They asked me how to make an appointment because someone in their family worked in a hospital, so it was easier for them to make an appointment, and they asked me if I would make an appointment. At the time, I hesitated.

A: Have you discussed the virus itself—how it spreads, the likelihood of infection, or infection rates?

B: We've talked about that already, but we mainly focus on maintaining hygiene in our daily lives. We're not particularly anxious about it happening because once we understood the specific transmission routes, we didn't dwell on it much.

A: Alright, aside from classmates and friends, you've also discussed this topic at home, correct?

B: Yes.

A: You mentioned talking with your mom. What did you discuss specifically—side effects, how to schedule appointments, or other infection pathways?

B: I discuss everything with my mom, from how to make appointments to asking about teachers, or rather, doctors. We covered what could happen, potential side effects, shelf life, and various other aspects. We also touched on transmission routes. Based on these, I explained to my mom that my risk of infection was already quite low and that the cost-effectiveness of the vaccine wasn't high, so combining these factors, I decided not to get vaccinated.

A: You're aware that the virus can spread through channels other than sexual contact. Despite considering the risks minimal, you feel it's not worth the effort, thus lowering its cost-effectiveness.

B: Yes, because I don't share items with others, avoid public spaces, and maintain cleanliness. I use my own things regularly, including personal items, which reduces the risk of infection even in private areas.

A: I see. So, with classmates, friends, and family, do you initiate conversations about getting vaccinated, or do these discussions often arise after someone mentions they got vaccinated?

B: Both happen?

A: You also proactively engage in discussions with others about the challenges of obtaining vaccines, for example?

B: Yes, during a period when vaccines were scarce, it was all over the news and a common topic among friends, especially among girls. It naturally led to discussions on this topic.

A: Now that the peak interest period has passed, have you noticed any changes in your mindset or in the frequency of discussing these topics with others?

B: We hardly discuss it anymore.

A: And you're not particularly focused on this issue anymore?

B: That's correct.

A: You've gathered a lot of information, and now you feel you know enough and don't need to look into it further, right?

B: Exactly. Actually, regarding hesitancy, I'm quite clear overall. My reluctance isn't particularly strong; I'm just not inclined to get this vaccine. As for concerns, as mentioned earlier, there are three points. Additionally, if you're interested, I can selectively discuss another topic from my fourth segment. About the stigmatization of the HPV vaccine, let me first explain what stigmatization means. Have you come across posts online that stigmatize the vaccine, particularly among older generations who might view it as spreading negative impressions about sexual behavior? Have you encountered such posts online, where people are troubled by these perceptions?

B: Yes, I know some people hold these views, but I personally haven't encountered anyone troubled by them.

A: Where did you come across these ideas? Was it on platforms like Zhihu?

B: It was on Douyin.

A: How do they express these ideas?

B: They would say things like, "It's embarrassing to say, but you're not chaste. You contracted a disease, so now you're getting injections," and so on.

A: Have you visited their profiles to see who's making these comments?

B: I've looked at some but not all. Many of these posts are from users who express doubts or ask questions similar to what's commonly shared.

A: They're not necessarily from the older generation; they could be from other demographics?

B: They're middle-aged men from the older generation. Just yesterday, I saw a post where a girl occasionally likes to drink alcohol. She hasn't done anything wrong, but a man she was set up with noticed she enjoys drinking based on her social media posts. When the girl ignored him, he said, "Let me tell you, stop drinking. If you drink, I'll tell you—you're like that." In reality, this man thought I was crazy and said, "Let me tell you, I have a friend who's a girl. When you drink, he doesn't want to go out."

A: When it comes to discussing infection pathways that involve sexual contact, do you ever feel it's intrusive to discuss such matters?

B: Personally, I think the intimate process itself is quite private. However, regarding issues like infections or other aspects related to sexual health, I believe those are suitable topics for discussion.

A: Do you think discussing HPV infections, for example, is something that can be openly talked about, or is it still considered taboo?

B: I think it's important to discuss. It can educate more women about practicing safe sex, emphasizing various precautions to ensure both partners' safety and personal hygiene. Because men can carry infections without showing symptoms, they can unknowingly pass them on to women, causing distress. By sharing information on transmission routes, more women can become aware of the risks and take preventive measures to avoid contracting the disease.

A: Do you feel there's an overall societal attitude that tends to overlook women's health issues like this?

B: Yes, I think there is. Men, in particular, might only think about themselves and not pay much attention to women's health or concerns about contracting diseases.

A: After initially researching extensively but now reducing your searches, do you ever feel fatalistic—like what's meant to happen will happen, regardless of whether you take preventive measures?

B: I did feel that way initially. I saw instances where people didn't take precautions yet didn't get infected, making me think, "Maybe it's just fate."

A: Do you ever experience an optimistic bias, believing that even if a disease is widespread, it won't affect you?

B: I do think it won't affect me. Even if it does, HPV can clear up on its own, and it's not a significant issue. It's like catching any other illness; it can be treated.

A: Why do you think many people hold this belief that it won't happen to them?

B: Based on my scientific analysis, I think the likelihood of contracting it is low, but nothing is certain. There's always a slight margin of error despite taking precautions.

A: Moving on, after reviewing so much information, do you think HPV has been unfairly demonized?

B: Demonized?

A: Yes, like the perception that everyone must get vaccinated, or else they're guaranteed to get sick. Has this been exaggerated?

B: It seems that way. Initially, my family thought it was a problem, but our interview was quite simple. I think I've covered everything; do you think it's useful?

A: This is based on my research.

A: Let me take a look. Basically, it has some points that can be used in my interview. You have a very useful point. It's that you've made a mistake. It can only prevent it for a year, but the protective time period, although research has yet to be finalized, but now, generally, it's 9 to 10 years.

B: 9 to 10 years, yes.

A: Why did you search for so much information, and you didn't see the real period of protection?

B: I don't know. Maybe my information-gathering skills are too poor.

A: No?

B: I don't know.

A: Actually, Baidu directly searched for HPV virus, and it had a Baidu column, Baidu health. Then it had a lot of information, and it also talked about it. Even though there is no precise research on the protection period in the clinical practice, there is a certain period of protection, 7 to 8 years, 9 to 10 years. Certainly, it is not just one year, because it is not impossible.

B: I don't know if this is one shot. At the time, it wasn't continuous. They said it wasn't continuous, but it was spaced out to take one shot, and then a total of three shots were taken.
