# Supplementary material for: Understanding cultural perceptions of sexuality in China and their influence on human papillomavirus vaccine hesitancy
Source: Front Public Health. 2025 Jan 23;12:1462722. doi: 10.3389/fpubh.2024.1462722 (PMC11801254; doi:10.3389/fpubh.2024.1462722)
Supplement: Supplementary file 1 [file Data_Sheet_1.zip › Frontiers_Supplementary_Material/Interview Transcripts - Participant 2.docx]

**Interview Transcripts - Participant 2**

A: Where did you first hear about the HPV vaccine?

B: The first time I heard about the HPV vaccine was probably online. I saw it on the Xiaohongshu platform.

A: What kind of information did you receive at that time? Did it tell you what the vaccine is for or anything else?

B: There is a lot of HPV information on Xiaohongshu, such as encouraging women to pay attention to their health. They often mention the HPV vaccine, provide tutorials on how to schedule an appointment, and explain the best times to get vaccinated. They also detail the precautions to take before and after vaccination and discuss potential side effects.

A: So, you mainly learned about this from Xiaohongshu. Have you also gathered information from other channels or sources about the HPV vaccine?

B: I mostly got my information from the internet, such as Weibo and Xiaohongshu. These platforms push this information to me when I browse. I rarely discuss this topic with people around me; my friends and I don’t usually talk about it.

A: Have any friends ever mentioned that they got the vaccine and shared their reactions with you? Or have any family members or relatives talked to you about the HPV vaccine?

B: No, my relatives haven't talked about it either. My family just says that without sexual activity, this topic doesn't come up much.

A: So, when discussing with your family, do you find it awkward to talk about sexual health?

B: It feels a bit awkward, even though there’s nothing to hide from them.

A: Okay. So, you mainly get information through online sources. Based on what you know about the HPV vaccine, how willing are you to get vaccinated? Do you have any hesitations or concerns?

B: I’m inclined not to get vaccinated. Although vaccination can be a protection for health, the side effects, especially after the COVID-19 vaccine, make me wary. I’m worried about the adverse effects on my body. There are also people who never got vaccinated and are fine. If the vaccine causes side effects, I would be more concerned about that. Like with the COVID-19 vaccine, you can still get infected after vaccination, and there are side effects. Many people have experienced nodules and other issues after infection. On Xiaohongshu, many people talk about the side effects of the HPV vaccine, which makes me even more hesitant to get vaccinated.

A: You mentioned earlier that you are hesitant to get vaccinated mainly because of the fear of side effects. Do you feel that balancing the potential side effects against the possible long-term benefits makes you more inclined to avoid the short-term side effects?

B: Yes, I feel that the side effects might outweigh the positive effects for me.

A: You also mentioned that you think not getting vaccinated won't be a big issue. Is it because no one around you has had this disease, or you haven’t seen many cases online?

B: I haven't encountered anyone around me with this disease, and I rarely see such cases online.

A: So, it's more common to see people getting vaccinated rather than reading about those who have contracted the disease?

B: Yes, and it used to be hard to schedule a vaccine appointment, but now it’s easier.

A: When many people were scheduling their vaccinations, did you feel the need to book an appointment too?

B: During that time, because of the pandemic, vaccines were scarce and hard to schedule. I heard about a friend who traveled across provinces to get vaccinated. I was in college in Lanzhou, and my friend flew to Beijing to get vaccinated. It required precise timing, and during the exam period, he had to make excuses to the teacher to go. Given the scarcity and difficulty in scheduling, I didn’t feel the urge to get vaccinated.

A: You mentioned earlier that the COVID-19 vaccine also influenced your decision. Have you had any other vaccines, and did any negative experiences with vaccines make you hesitant?

B: I haven't had other vaccines recently. Most were given when I was a child. As an adult, I feel my immunity is stronger, and I’m more worried about potential side effects from new vaccines.

B: Like the COVID-19 vaccine, which might not be fully developed, leading to minor health issues.

A: So, the COVID-19 vaccine has significantly impacted your willingness to get the HPV vaccine.

B: Yes, but I might be willing to get vaccinated in the future.

A: You’re not entirely against getting the HPV vaccine in the future, right?

B: Correct, I'm not against it.

A: What factors might prompt you to get the HPV vaccine in the future?

B: As more people talk about it, and considering health issues, I might consider it. Especially if I live with or have close contact with a partner, which increases the risk of these infections. This disease seems to be becoming more common among younger people, so vaccination would provide some protection. Currently, I have some resistance due to the COVID-19 vaccine, but I’m not completely against getting it if the timing is convenient.

A: You mentioned that living with a partner might be a factor. Do you think there’s a connection between getting the vaccine and starting a sexual relationship, and are you worried about how others might perceive you for getting the vaccine?

B: I don't think it matters how others see it. It's about protecting myself, and their opinions are not as important. I wouldn't compromise my health based on what others think.

B: I won't be influenced by others saying I shouldn't get vaccinated, nor will I avoid it because of someone else's opinion. I don't care much about others' views.

A: Earlier, you mentioned that you got most of your information about the HPV vaccine from online sources. Do the opinions of others online influence your decision?

B: Yes, if people talk a lot about it, it can be like an explosive spread of information in front of you. If many people emphasize the importance and benefits of the vaccine, I might feel more inclined to get it. But if others talk about side effects and advise against it, I might feel more hesitant. I am quite influenced by what people say online.

A: In your past online interactions, have you come across more positive or negative information about the HPV vaccine?

B: I think I usually see more positive posts, encouraging people to get vaccinated at the right age and explaining the benefits. There’s a lot of promotion about the different types of vaccines, like the nine-valent one. However, in the comments, there are often debates, with some people arguing against the benefits and discussing side effects. Overall, I think there is more positive information.

A: So, there is more positive information than negative. Which type of information influences you more?

B: I think I am more influenced by the positive information. I tend to be optimistic and believe things will get better. Since the vaccine is meant to ensure health, I believe it is mostly good. Negative cases are probably rare, and most people only report minor discomfort like a sore arm after vaccination. In the long run, vaccines seem to benefit health.

A: So, you’re not particularly affected by negative information online. Your vaccine hesitancy is more influenced by personal factors, especially your experiences with COVID-19. Is that correct?

B: Yes, recently I’ve been sick for a long time, and hospital visits revealed minor issues. I feel these might be related to vaccines or cold weather, and now I am more resistant to vaccines, though I was more open to them before.

A: I see. Do you think the HPV vaccine might be influenced by cultural or moral factors? For example, people might worry about being asked about their sexual history by doctors or think that being chaste means they don’t need the vaccine.

B: Some people might think that discussing HPV vaccines or getting gynecological exams implies promiscuity, but I haven’t encountered such views. For me, the HPV vaccine is just like any other health vaccine, ensuring one’s health. Getting vaccinated doesn’t imply anything about one’s moral conduct. If someone already has HPV, the vaccine won’t help. So, I don’t see significant moral or cultural constraints.

A: If, in the future, authorities or institutions promote the HPV vaccine like they did with the COVID-19 vaccine, providing it for free, would that significantly influence your decision or reduce your hesitancy?

B: I might be slightly resistant to such widespread campaigns. Although wide availability suggests safety, I worry it might have flaws like the COVID-19 vaccine. The HPV vaccine, especially high-quality ones like the nine-valent or imported versions, is costly. I feel more assured paying for a vaccine than receiving a free one, which might not use the best materials.

A: Okay. This interview has covered your understanding of the HPV vaccine, reasons for hesitancy, and cultural influences. It seems that your main concern stems from negative experiences with COVID-19, rather than cultural or moral constraints. Is that correct?

B: Yes, that's correct.

A: Great. This concludes our interview. Thank you very much for your participation.

B: Thank you. Goodbye.

A: Goodbye.
