# Supplementary material for: Understanding cultural perceptions of sexuality in China and their influence on human papillomavirus vaccine hesitancy
Source: Front Public Health. 2025 Jan 23;12:1462722. doi: 10.3389/fpubh.2024.1462722 (PMC11801254; doi:10.3389/fpubh.2024.1462722)
Supplement: Supplementary file 1 [file Data_Sheet_1.zip › Frontiers_Supplementary_Material/Interview Transcripts - Participant 3.docx]

**Interview Transcripts - Participant 3**

A: First, let's talk about the virus itself, HPV, which is called the human papillomavirus in Chinese. Could you tell me what you know about this virus?

B: Honestly, I don't know much about it. I remember that the vaccine hasn't been around for many years. People say you should get vaccinated to prevent it, but it's hard to get an appointment and it's not cheap. My friends say it's necessary, but I don't really understand what it's preventing. It seems like it's something women need to guard against, so I think I don't know much about it. I know very little.

A: I'll ask specific questions, and if you don't know, just say so. How do you think the virus spreads? Have you seen anyone around you infected with it, or have you seen posts online about people getting this virus?

B: I haven't heard of anyone around me getting it, but I've seen posts online suggesting that it's recommended to get the vaccine as soon as possible.

A: Are those posts from people who have been infected and are urging others to get vaccinated, or just general recommendations?

B: It's more like general education. They talk about the risks for women and the importance of getting vaccinated.

A: So, no one specifically saying they've been infected with HPV?

B: No, I haven't heard of anyone like that around me.

A: Alright. The next question is, how do you think HPV is transmitted?

B: From what I know, it's mainly through sexual activity. I'm not sure about other ways.

A: That's fine. If you don't know, that's okay. Do you know what diseases an HPV infection can lead to?

B: Diseases from this virus? I've heard it can cause some gynecological diseases and possibly some cancers, like cervical cancer.

A: Yes, cervical cancer.

B: Right, and other serious cancers. That's what I've heard, along with some other gynecological diseases.

A: Who do you think is more likely to get infected with HPV? For example, people with weak immune systems are more likely to catch a cold. What about HPV?

B: I think people with more chaotic sexual lives and poor hygiene are more likely to get infected. I've also heard that people with weaker immune systems might be more susceptible.

A: Okay, that's about the virus. Now let's talk about the vaccine. You mentioned earlier that you don't know much about the vaccine. Do you know what types of HPV vaccines there are?

B: I don't know the types, but I know the price range. My friends have told me it's around 1,000 yuan per shot, and it usually requires three shots.

A: Yes, the 9-valent vaccine costs over 1,000 yuan per shot and requires three shots. Do you know the recommended age range for getting the HPV vaccine?

B: My friends told me to get it quickly before turning 24 or 23, so I think the age range is around 23 or 24.

A: Do you know how long the protection from the HPV vaccine lasts?

B: No, I don't know.

A: That's okay. You mentioned your general feeling towards vaccines earlier. Can you elaborate on that?

B: I feel like vaccines we got as kids, like for chickenpox, protect us from those diseases for life. But with the COVID-19 vaccine, I realized that it doesn't provide complete protection, just reduces the risk. So, I think vaccines mainly reduce the possibility of getting a disease but don't completely prevent it.

A: Yes, many people have mentioned the impact of the COVID-19 vaccine on their perception of vaccines. Based on your understanding of the virus and the vaccine, how hesitant are you about getting the HPV vaccine?

B: I'm not very hesitant. I just don't feel a strong need to get it right now. The cost is a factor since it's expensive, and I'm a student. Also, since it's mainly transmitted through sexual activity and I don't have that activity, I feel it's not urgent for me. I might consider it later when I get married, but then again, the age limit is 23 or 24, and I'm nearing that age. So, overall, I don't feel it's very necessary for me.

A: Let's break this down. First, the cost. Do you think it's worth spending money on the vaccine compared to other things you might spend money on?

B: I don't think it's worth it because I don't see it as a necessity. If I need a new computer or phone, I would prioritize spending money on that instead. The vaccine seems like an extra expense that's not urgent for me.

A: Where does your money come from for big purchases? Do you usually rely on yourself or ask for help from your family?

B: Mostly from my family. I'm still in school, so my income is limited to some allowance and occasional small payments from school work.

A: You mentioned not needing the vaccine because you don't have sexual activity. Have you considered that HPV could be transmitted through other means?

B: If it were transmitted through more common ways like the flu, through the air or saliva, I would definitely consider getting vaccinated. But since it's not, I don't see it as necessary.

A: There's a notion that vaccines should be taken before exposure to the virus, similar to the COVID-19 vaccine. Do you know that the HPV vaccine should be taken before sexual activity begins?

B: Yes, but since I don't have sexual activity now, I feel it's not urgent. If I had such behavior, I might consider it more seriously.

A: Your main source of information about the virus and vaccine—do you get more information online or offline?

B: Almost all my information comes from online sources. People rarely talk about these things face-to-face. Online, it's easier to find information and discuss these topics.

A: What kind of information have you found online about HPV and the vaccine?

B: Mostly about how difficult it is to get an appointment for the vaccine and that it's important for women. I haven't seen much about side effects, except for one time my roommate had a fever and wondered if it was related to the vaccine.

A: So, the information you get is mostly about the difficulty of getting the vaccine, which is somewhat discouraging?

B: Yes, it feels like a hassle to me.

A: Do your classmates talk about the vaccine often?

B: Not really. They might mention it when they've managed to get an appointment, but not in-depth discussions about the virus or the vaccine's effects.

A: Have your family members ever brought up the topic of the vaccine?

B: No, my family hasn't mentioned it at all. I don't think they even know about it.

A: You mentioned that discussions about sexual activity and related diseases aren't common among your peers. Why do you think that is?

B: I think it's because people aren't comfortable discussing such personal topics unless they have a close relationship. It's easier to talk about general aspects of relationships but not about sexual behavior and related health issues.

A: Lastly, let's talk about possible misconceptions about the vaccine. Some older generations might associate the HPV vaccine with promiscuity or negative sexual behavior. Would such views affect your decision to get vaccinated?

B: Not really. If I heard such opinions, I would just think they're uninformed. I believe in making my own decisions based on accurate information.

A: Some parents are hesitant to vaccinate their children, fearing it might encourage sexual activity. If your parents had similar concerns, would that influence your decision?

B: No, because my parents usually trust my judgment. Even if they had concerns, I would explain the importance of the vaccine to them and make my own decision.

A: Thank you for sharing your thoughts. This concludes our interview.
