# Supplementary material for: Understanding cultural perceptions of sexuality in China and their influence on human papillomavirus vaccine hesitancy
Source: Front Public Health. 2025 Jan 23;12:1462722. doi: 10.3389/fpubh.2024.1462722 (PMC11801254; doi:10.3389/fpubh.2024.1462722)
Supplement: Supplementary file 1 [file Data_Sheet_1.zip › Frontiers_Supplementary_Material/Interview Transcripts - Participant 4.docx]

**Interview Transcripts - Participant 4**

A:What do you know about HPV vaccines? Can you talk about when you first heard about HPV vaccines?

B:I think I first heard about HPV, probably on Xiaohongshu or when people around me started scheduling appointments for it. My understanding is that it prevents various HPV infections, including preventing cervical cancer. However, I haven't delved deeply into it myself. If I come across information on Xiaohongshu, I might take a look.

A:Do you know, for example, how HPV infects people or why people get infected with HPV?

B:From what I understand, it's mainly transmitted through sexual activity, which can lead to HPV infections. I haven't looked into it much further on my own.

A:Do you take specific measures to prevent HPV infections in your daily life?

B: Because I've seen a lot about it on social platforms, and in some public spaces, including when I was interning and sharing a place with male roommates, I've become a bit cautious. For instance, I would disinfect common items in the bathroom, which makes me a bit concerned. So, my attention to this is somewhat heightened.

A:Alright. Based on what you've learned about HPV vaccines, how willing are you to get vaccinated?

B:On a scale of 1 to 10, right?

A:Yes, please rate it.

B:I would say 6 to 7.

A:Hesitant between 6 and 7? Or is your willingness 6 to 7?

B:I'm somewhat hesitant.

A:Next, let's talk specifically about the reasons for your hesitation.

B:Sure. First of all, one reason is my own situation. It's been quite a few years since I've been in a relationship, so considering I'm not currently sexually active, I feel the concerns or risks in this area are relatively low right now. Therefore, I think it might not be necessary for me to get vaccinated at the moment. Secondly, economically speaking, as I'm still a student, my parents aren't very familiar with vaccines or preventive measures. I've talked to my mom about this vaccine before, but after she listened, she probably didn't take it as seriously as young people do nowadays. She casually mentioned that if I want to get vaccinated, she could provide financial support. However, from my perspective at the time, if they weren't strongly supportive or had an ambiguous attitude toward it, I might lean toward paying for the vaccine myself, considering my studies and future earnings. Thirdly, because the requirements for his age aren't as strict as before, and the appointments aren't as difficult to schedule as they used to be. I remember there was a time when the nine-valent vaccine was particularly hard to get, and people might even find others to queue for them. But now it seems like there's enough supply, so I think I can get vaccinated whenever I want. So, primarily, I think it's because I haven't been in a relationship, which isn't particularly relevant to my current situation. The last reason is actually because of the discussions I've seen on social platforms about HPV. Previously, everyone encouraged getting vaccinated, but now I see some negative reactions after vaccination, like some people saying their immune systems weakened or they became more prone to allergies. So now I'm taking a more cautious approach. Whereas before, I thought it was good, now I'm wondering about its actual effectiveness and whether these adverse reactions really exist. And if they do, whether they might have some impact on my immune system, which is also quite important. So, those are the points of concern for me.

A:You mentioned earlier that although your hesitation is high, it doesn't mean you won't get vaccinated at all. What do you think will eventually persuade you to get the HPV vaccine?

B:I think it might be when I enter a stable relationship or, to put it simply, if I foresee having a sexual life in the near future. I would definitely consider getting vaccinated before that or during that time. Because these issues are often reported in the news and are quite common, saying one isn't worried at all isn't very realistic. So, if I have such intimate relationships, I will consider it.

A:There were discussions online that if you have a stable sexual partner, there's no need for the vaccine because the chances are low if you're only with one person. What do you think about this view?

B:I actually have a relatively neutral attitude towards this because I think relationships between people can change quickly. Also, seeing so many stories about guys online, I think even if you have a stable boyfriend, you don't know what he might do outside or who he might contact. So, I don't think it's very realistic to rely solely on trust. It's probably more about self-protection. Secondly, even if you think it's a stable relationship now, there may be changes later. Once you start, it's probably better to be cautious.

B:Because I often hear from male friends that going for massages or visiting prostitutes is quite common, so even if it's a stable relationship, my trust in him wouldn't be that high, and I think it's important to protect myself.

A:Exactly. Besides this point, you also mentioned an economic factor earlier, and you also mentioned family reasons. Perhaps the viewpoints of our parents' generation differ from ours. Why do you think mom and dad might not place much importance on this matter, and what's their thought process behind it?

B:Firstly, it may indeed be due to different environments they grew up in. During their time, such things didn't exist, and their concern for women's health wasn't very high. They would handle things after they happened rather than thinking ahead for prevention. It's definitely a matter of mindset. They may not have the same awareness of preventive measures as our generation does now. Secondly, it might be due to the limited information they receive, including their cultural background. We get a lot of information from the internet nowadays, even about the nine-valent vaccine, and people around us are more open to discussing sexual topics. But for our parents' generation, they probably have less exposure to such information online and aren't proactive in learning about these topics. It might also be based on their cultural background or their living environment. They might not come across information about this, and their understanding of it isn't sufficient. Thirdly, I think it's a matter of mindset. They might not place enough emphasis on women's health themselves. Even though they know about the vaccine's existence, they might not see it as necessary. There are many posts on Xiaohongshu that are similar to my situation. You try to introduce the HPV vaccine to them, but based on your explanation alone, they can't really feel its importance. They still don't take it seriously enough.

A:Good. In your earlier hesitation factors, you mentioned economic considerations. I'd like to explore your value judgment. For instance, university students may not have a lot of money, but they might still spend around 4000 RMB on the HPV vaccine (9-valent), with 2-valent and 4-valent being slightly cheaper. Sometimes, I feel spending 4000 RMB on travel is very worthwhile, but when it comes to spending money on vaccines, it feels like it's not worth it due to certain factors. Could you talk about your decision-making process?

B: Sure, my value judgment usually depends on recent or recent events within a timeframe. As I mentioned earlier, one of the main hesitation factors is that I currently don't have a romantic relationship, so this matter isn't urgent for me. During my internship, I had some savings and plenty of time and money to schedule and get vaccinated. However, at that time, I felt this matter was still somewhat distant from me, and I preferred to focus on completing more immediate goals, like traveling or using the money for other personal matters. So, my value judgment usually leans towards what's more important in the current period, rather than focusing on longer-term goals like saving for my future life a year or two from now.

A: I see. It's about balancing short-term versus long-term perspectives.

B: Exactly.

A: Yes, leaning more towards short-term. College students often prioritize immediate pleasures like eating, drinking, and entertainment. Long-term considerations are put on hold.

B: Right, because for me, even before the age range was extended, the age range for the 9-valent vaccine was only up to 26 years old. Now, university students may not be that urgent in terms of age, so there's no immediate rush to get vaccinated.

B: Because for me, if I were sexually active during my university years, I would definitely consider getting this done as soon as possible. But since it's not urgent for me, I feel it's not necessary at the moment. Personally, I may be more concerned about personal hygiene aspects, which I see more frequently online.

A: I see. Let's discuss the last point you mentioned about the perception of HPV. You mentioned earlier that there used to be more information online encouraging people to get vaccinated. Did you feel influenced by that at the time?

B: We were influenced to some extent. Because for a period of time, it was quite common to see people who quickly shared their experiences with the HPV vaccine on social platforms, and they would even post photos of the vaccination process. At that time, it was probably because it was the first time you were exposed to this thing, and you didn't know exactly what it was. Seeing everyone else getting vaccinated, you might feel the urge to follow suit, which I think influenced my judgment to a certain extent.

A: And why didn't you follow everyone else and get vaccinated? What were your considerations?

B: At that time, it was probably because I was still a graduate student, and it was probably due to economic reasons. I didn't really want to directly ask my family for money to get vaccinated.

B: So the degree of following the trend wasn't enough to make me think of it as a priority to ask my parents for money to get vaccinated.

A: When did you feel the trend changed?

B: I think it started to change over a year ago, maybe around the beginning of 2022 or 2023. Because before that, I basically didn't see much about any adverse reactions, but later, when the pandemic was at its worst and people were feeling anxious, there were some posts about people feeling that their immunity wasn't as good as before after getting the HPV vaccine. There were quite a few such posts on Xiaohongshu (a Chinese social platform). And then there were also some people who didn't mention any adverse reactions, but they began to wonder if it was effective after they got vaccinated. Since they didn't have a stable intimate relationship at the moment, they felt it was still quite distant for them. So from what I've seen, these discussions have focused on these two aspects. There are fewer posts encouraging everyone to rush and get vaccinated, and relatively speaking, people are now in a more rational or objective stage. Of course, it may not be entirely objective, but it's probably less impulsive or trend-driven than before.

A: I see. In your previous answers, you mentioned that some people were influenced by the COVID-19 factor. Did you feel the impact of the previous COVID-19 vaccine?

B: Personally, I didn't feel a significant impact from the COVID-19 vaccine. I only experienced some temporary allergies after exposure, but later on, I didn't feel that the vaccine had any other effects.

A: Okay, good. You mentioned earlier that you tend to gather more information online than offline, as mentioned before. Your family has also been mentioned. Have you discussed any information about the vaccine with your classmates or friends?

B: I have discussed this with my friends. But our discussion mainly revolves around whether men should take the initiative to approach HPV. Because during our discussion, we felt that there was some risk from men. And in China, a common belief is that women are more likely to get vaccinated. But my friend studied abroad, and she came back saying that in foreign countries, male vaccination is more common. After they are vaccinated, the protection for women may be higher than if women were vaccinated themselves. So we might discuss this issue and suggest to our male friends whether they should also get vaccinated, in order to protect their future girlfriends. It's a collision of ideas.

A: Some friends of mine have mentioned this to me too. They feel that while it's optional for girls, boys must get vaccinated because if ...

B: My friend suggested that since male vaccination is more common abroad, she would advise my male friends when she returned, saying that if they have time, they can get vaccinated. Because in their case, male vaccination may be more effective. I don't have a deep understanding of the specific situation.

A: So you didn't convince them?

B: We did. Actually, I think most of them didn't explicitly refute it, but they didn't take action either. But one of my friends said he would consider it.

A: When someone says they'll consider it, it usually means they've taken it to heart.

B: Yes, he did take it to heart, but I think for most of my male friends, they didn't fully accept it. They may just listen when we talk about this topic and introduce it to them. Their concept is still in the stage where they see only girls getting vaccinated, so they may be more willing to spend money on their girlfriends' vaccines rather than spend money on themselves to get vaccinated.

A: Dealing with it from the source. I think getting vaccinated is really loving your girlfriend, which also counts as protecting your girlfriend.

B: I think male vaccination is also a topic worth discussing.

A: My main interview covers these aspects. Later, I may briefly discuss whether you think the HPV vaccine still relates to some of our cultural traditions, or whether it is subject to cultural restrictions because people don't talk much about sex.

B: I think it does. Even though many people get vaccinated, they don't necessarily discuss how the virus itself infects people. They only know about its protective effects and choose to get vaccinated, while discussions on other topics are less frequent. That's my perspective based on the vaccine itself.

And secondly, I think traditional concepts are more restricted by parents. Because for them, sexual relations and sexual education may be influenced by cultural factors, leading them to attach less importance to knowledge or attention in this area. So when you discuss this topic with them, they won't feel a great impact. And of course, like me, I definitely won't talk to my parents openly and honestly about some sexual relationships or sexual knowledge. So I think this influence exists, right.

A: At that time, I mentioned to my mom that there's a vaccine to prevent cervical cancer, but she didn't know much about how it's transmitted. Did you also educate your parents about this?

B: I didn't go into detail with my mom about how HPV infection occurs. I just mentioned briefly that everyone is getting vaccinated now, and it can prevent cervical cancer and HPV infections. I didn't have a detailed conversation with her about it. I mostly sensed her attitude towards it, you know? I felt discussing these things with them might be a bit uncomfortable.

A: Also, the financial aspect comes into play. They might not understand spending several thousand yuan on a vaccine, and if they did decide to get vaccinated at this stage, they might contribute financially, but it's hard to have a deep conversation with them about it.

B: Unless they're personally very concerned about it, I wouldn't delve deep into the topic with them.

A: With friends and classmates, we mostly discuss how to get vaccinated or any side effects. We rarely discuss the virus itself and how it infects people.

B: Right, that's quite common. Among friends, you might ask how they managed to get an appointment or how they felt during the vaccination.

A: And maybe we wouldn't talk about what symptoms they had after infection, or how they got infected, or what precautions they should take in their daily lives. These topics are rarely discussed.

A: Actually, university students might be more open, but they still don't discuss their personal lives in this aspect much, right?

B: Maybe the first aspect is about the level of privacy individuals want to maintain, which also depends on the depth of their relationships. Normally, friends won't talk about the intimate details of their relationships. The second aspect, even though there's openness, the overall atmosphere is still quite traditional. Moreover, due to the lack of sex education in early education, it's not a topic they're comfortable discussing. This is probably the result of early education.

A: I agree. So, if someone goes to get vaccinated later, and the doctor asks about their sexual experience, would they find it awkward, or are they already in a mindset where they accept it normally?

B: Personally, I wouldn't be very comfortable discussing this topic in great detail with the doctor, but I wouldn't feel extremely awkward either. I think I could relax and talk about my current situation in this regard, because after all, it's for my own health, and the doctor wouldn't be too concerned about it.

A: Right, we also mentioned earlier that some parents of older generations don't pay much attention to sex education, and some may even stigmatize it. For example, they might think that abstinence can prevent the need for vaccination. Do you see any connection between these views? Or how do you view these viewpoints?

B: I think these views are influenced by traditional and even feudalistic thoughts, because historically, in relationships between men and women, men were often seen as "clean" while women were often blamed for any issues in sexual relationships. This is still a prevalent mindset among older generations. They may believe that if girls keep themselves clean and refrain from promiscuity, they won't get infected, but in reality, some girls are in stable relationships without knowing what their partner has done. I think these more feudalistic views and concepts affect why some still advocate for girls to remain pure.

Secondly, traditional views on romantic relationships or marriage may also play a role, as many parents still don't support premarital sexual behavior.

A: Yes, there are views suggesting that giving your child this vaccine means encouraging them to start having sex, or something similar.

B: Some parents are too conservative to accept this.

A: Exactly. In my literature review, I found research on hesitations among parents of middle school students regarding HPV vaccination. It discusses their concerns that vaccinating their children might imply they will engage in sexual activities.

B: Ok, it seems they're not quite open-minded yet, thinking that vaccination automatically leads to sexual activity, without considering prevention.

A: Right. Actually, that concludes the discussion part of my interview.
