# Supplementary material for: Understanding cultural perceptions of sexuality in China and their influence on human papillomavirus vaccine hesitancy
Source: Front Public Health. 2025 Jan 23;12:1462722. doi: 10.3389/fpubh.2024.1462722 (PMC11801254; doi:10.3389/fpubh.2024.1462722)
Supplement: Supplementary file 1 [file Data_Sheet_1.zip › Frontiers_Supplementary_Material/Interview Transcripts - Participant 5.docx]

**Interview Transcripts - Participant 5**

A: What aspects of information do you currently understand about HPV vaccines?

B: It prevents the occurrence of certain cancers and sexually transmitted diseases for women. It's a vaccine that prevents some cancers and STDs. Besides that, there are other pieces of information...

A: Do you remember where you first heard about the HPV vaccine?

B: It was on Weibo. I saw people going to get vaccinated, and at that time, I didn't understand what the vaccine was. So, I searched it up on Baidu.

A: Did you have thoughts back then whether you wanted to get vaccinated, were indifferent, or didn't want to get vaccinated?

B: At that time, I wanted to get vaccinated. But people were complaining about how difficult it was to make appointments, and when I checked, it was indeed challenging. Also, I didn't know much about the channels for booking appointments, and the cost was quite high, over 3000 to 4000 RMB. So, I wanted to get vaccinated but didn't know how to start.

A: Okay. Do you know specific information about HPV, like why someone would be infected with the HPV virus?

B: For women, it could be through sexual contact or in public environments where there might be bacteria, like swimming pools or hotels with shared items. You could get infected by contact.

A: Have you ever purposely taken precautions to prevent infection by this HPV virus?

B: Because before, I didn't know that swimming pools could transmit it. I used to think it was only transmitted through sexual behavior. Recently, I learned that it could also be transmitted through these public facilities, so I used to think that if I didn't engage in such behavior, I might not need to be concerned. But after learning this, I didn't specifically take precautions during swimming lessons because I wasn't very clear about it at the time.

A: Based on your understanding of basic information about HPV and vaccines, how hesitant are you currently about getting vaccinated? On a scale of one to ten.

B: Hesitation? About seven.

A: So, you're still in a relatively hesitant state.

B: Because it's said to be best to get vaccinated before age 26, and there's a time limit. But it's very difficult to make an appointment, and you might have to wait in long queues, especially at school. If you're not at school, you don't know when you'll get vaccinated. Also, in our area, I searched before, and it seems that there's no way to make an appointment. So, I've been very hesitant. Also, my family doesn't know about this vaccine at all, and I would hesitate to explain why I need to get vaccinated and how to talk to them about spending three to four thousand RMB on vaccination.

A: Besides the above factors—time, difficulty in making appointments, discussing with family, and economic factors—are there any other factors that cause you concern?

B: When you search online, they often mention side effects, like acne outbreaks or irregular menstruation, or even loss of menstruation. These potential side effects are concerning because you don't know when they might occur. Also, I haven't specifically seen how long the vaccine's protection lasts, so I don't really understand that. That's about it.

A: Indeed, official articles online also mention that even after getting vaccinated, it doesn't guarantee that you won't get infected. Regarding the factors you just mentioned, which involve difficulty explaining to your family or discussing economic limitations, is there anything else that influences your decision-making positively?

B: Besides preventing diseases, another positive factor is that it might help me integrate better with others because at our age, many girls are choosing to get vaccinated. So, if I get vaccinated, I might feel more integrated or have more topics to discuss.

A: Your viewpoint is very innovative. So, do you consider yourself to have a herd mentality?

B: Yes, it's a herd mentality because before I didn't understand it at all. But if others do it, I might want to understand why and realize I might need it too.

A: Like you mentioned earlier, it's too difficult to make appointments. Do you feel discouraged more by the difficulty, like I can't make an appointment, or does it stimulate a kind of mentality that everyone is making appointments, so I should hurry to make an appointment?

B: It's discouraging because it's very troublesome. I often see others, such as in other places, where they can get vaccinated for free, and some places even arrange vaccinations for girls in their teens. But here, maybe you have to call the community health clinic to make an appointment, and they tell you to line up. Or if you go to a certain organization to make an appointment, I've seen that they require you to first undergo a test costing several hundred RMB before they'll administer the vaccine. So, I think it's really troublesome, and it's also easy to spend a lot of money on things you shouldn't spend on.

A: Yes. So, you're currently in a state of hesitation, but it's not absolute that you wouldn't consider getting the HPV vaccine in the future, right? What might be a factor that could prompt you to get vaccinated?

B: If I could schedule an appointment immediately after making one, rather than having to wait in line for a long time without knowing when I can get vaccinated, that would make it more convenient.

A: So, it's primarily about convenience. The difficulty in scheduling appointments is more of a limiting factor, right?

B: Yes, because for instance, at my school, resources are relatively scarce here. If I were to schedule an appointment at school, by the time they call me back after half a year, I might not even be there anymore, which would be troublesome to figure out.

A: If you imagine a scenario where the vaccine is readily accessible to you—if you needed to get vaccinated and I could administer it to you right now—would you still hesitate?

B: Probably not. I would likely go ahead and get vaccinated if I've saved up enough money.

A: So, your main hesitation is more about the difficulty in scheduling rather than concerns like potential side effects or uncertainty about how long the vaccine protects, correct? Ok, understood. Moving on to how you gather information about the HPV vaccine—have you discussed HPV vaccine-related information offline with others, like classmates or friends?

B: Only on platforms like Weibo have I seen discussions where people mention getting vaccinated. It seems my roommates around me haven't brought it up or discussed it. I'm not sure why they're not aware or maybe they haven't brought it up. It seems like it hasn't been discussed with them.

A: People around you haven't discussed these things.

B: Right, I've learned about it online.

A: Including relatives and friends—have any medical personnel among them discussed HPV vaccine information?

B: No.

A: Regarding the channels you use online, are they mainly platforms like Douyin, Xiaohongshu, or WeChat?

B: It's mainly Weibo and WeChat. Also, in WeChat, there are classmates, although I'm not close with them at university. I've seen them post about getting vaccinated on Moments, but it wasn't a big discussion.

A: From the information you've seen online, what kind of information have you come across regarding the vaccine?

B: For example, on Weibo, I see people discussing which dose they're getting, and some mention experiencing pain or developing acne after vaccination. On Xiaohongshu, there are posts encouraging everyone, especially females, to get vaccinated. There are also discussions about which type to get—2-valent, 4-valent, 9-valent—and considerations about choosing between domestic and international options.

A: Do you find there's more positive information or negative information?

B: I see more positive information, but sometimes I also look into potential side effects and search specifically about them.

A: Even though there's more positive information, do you find yourself influenced by negative information to some extent?

B: Yes, I also look into negative aspects.

A: Ok. Do you personally initiate conversations about HPV with others?

B: I don't know why I haven't brought it up myself. I think I talk with my roommates about a lot of topics every day, including social issues occasionally, but it seems we haven't really discussed this.

A: We talked earlier about why people around you haven't mentioned or discussed this topic. Do you think this might be related to our relatively conservative culture in China? That is, does our conservative culture create a taboo around discussing these topics?

B: I think it's possible. I haven't really talked with my roommates about it. Sometimes, for example, my roommate might go out with her boyfriend and even spend the night out. I think it's somewhat dangerous for girls to do that. Maybe it's because I'm a bit traditional and feel that they shouldn't spend the night out, but I don't know how to bring it up.

A: Right. You just want to give a kind reminder, but even such a well-intentioned reminder makes it hard to discuss premarital sex, right?

B: Yes, that might be the case. If you hadn't mentioned it, I wouldn't have thought about discussing it with them.

A: Before, you haven't discussed sexual topics with people around you, aside from online platforms, right?

B: Yes.

A: Given this conservative culture, do you think it leads to the idea that if you conduct yourself well, you don't need the HPV vaccine? Or, if you get the HPV vaccine, would the older generation think negatively of you, assuming you're part of a sexually active group?

B: I think it might. If they don't understand, you could explain that it's just a cancer prevention vaccine, but if they find out what it's for, they might question why you got it. I do care about their opinions and wonder if they would think differently of me, even though I've never acted that way.

A: Right. Some of the older generation might avoid directly using the word "sex," opting for euphemisms. In my previous literature review, I found that some parents hesitate to vaccinate their children against HPV because they worry it might encourage sexual activity.

B: To be honest, I still don't know how to talk to my parents about it. Even seeing it on TV makes me feel awkward. My roommate mentioned feeling very awkward when a movie with such scenes came on while watching it with her parents. She didn't want to watch those kinds of scenes with them and didn't know how to deal with it.

A: We talked earlier about why people around you haven't mentioned or discussed this topic. Do you think this might be related to our relatively conservative culture in China? That is, does our conservative culture create a taboo around discussing these topics?

B: So it might influence us. If you want to promote it, you might tell people it's to prevent sexually transmitted diseases. But if you want to promote it here and have parents see it, they might find it hard to handle the promotional message on public platforms.

A: Sometimes educational efforts might go wrong. If they only see that it's to prevent sexual infections, they might think, "Why do you want the vaccine? Are you sexually active or spending nights out with your boyfriend?"

B: Yes, that's true. So the focus of the educational campaign is important. It seems like we don't see widespread public health campaigns on this topic.

A: From my interviews, I've found that many people get their information online. Offline, no one talks about it. Among classmates, it’s rarely mentioned, probably because of our conservative culture. People mainly learn about it online.

B: Yes, so I think we need to work on the promotion, but we need to think about how to do it effectively.

A: There's a deeply ingrained conservative mindset, making it difficult to change the older generation's views.

B: So it's a long and challenging task.

A: Yes, it is.

B: But I think the younger generation, like ours, is more open to it.

A: Right.

B: We might find it easier to discuss it with our elders.

A: Yes, in my interviews, I found that most university students don't find it hard to talk about sex. Some might be a bit conservative, but they generally don't see it as a difficult topic. However, discussing it directly with older people is much harder.

B: Yes, it's difficult. Maybe when our generation grows up, it will be a bit easier. We might choose to vaccinate our children when they are teenagers. I think our generation will do better in promoting this. For example, making the vaccine more available at universities so students can get vaccinated easily is a good idea.

A: Right, or promoting it to women in their mid-twenties who have some financial independence and understand the risks. They might choose to get vaccinated without involving their parents.

B: Yes, it's important to educate the youth because the older generation is slower to accept new information.

A: Besides the conservative mindset, some participants mentioned concerns about the impact of COVID-19 vaccines on their bodies, which might affect their willingness to get the HPV vaccine. Do you have any concerns about this?

B: Yes, definitely. For instance, when we got the COVID-19 vaccine, we were practically forced to get it, with the community calling us to ensure we did. Some people were hesitant because it was a newly developed vaccine, and no one knew its side effects or long-term impacts. I remember a well-respected, knowledgeable professor who refused to get the vaccine, which made me second-guess my decision after I had already been vaccinated. There were also rumors about various vaccines, and even though I tried to ignore them, they still caused some anxiety. Personally, I felt that my sleep was affected, although this might be just my perception.

A: Yes, I also felt that even after getting vaccinated, people still got infected with COVID-19, sometimes multiple times.

B: Exactly. The initial promise was that the vaccine would reduce the severity of the symptoms, not completely prevent the infection. But you couldn't really tell what the severity would have been without the vaccine. It created a lot of uncertainty.

A: There's a significant uncertainty factor, and the benefits seem minimal.

B: Yes, so after this experience, if there's a need for future vaccinations for other viruses, I might hesitate more and possibly choose not to get vaccinated.

A: You mentioned mandatory vaccinations earlier. If the government strongly promotes the HPV vaccine, would you still have reservations, or do you think encouraging HPV vaccination is acceptable?

B: I think it's acceptable because the HPV vaccine has been around for a while, and many people have received it. Seeing that others are getting vaccinated makes it seem more trustworthy.

A: You mentioned a herd mentality, feeling more inclined to get vaccinated when you see others doing it.

B: Yes, I think many people feel this way. Even if the vaccine isn't heavily advertised, seeing others choose to get it creates a sense of trust and willingness to follow suit.

A: That concludes our interview for today. We've covered some general questions and delved into cultural factors influencing attitudes toward the HPV vaccine. Thank you very much for your time and insights.

B: You're welcome. If you need any further information, feel free to contact me.

A: Yes, you can reach out to me anytime.

B: Alright, thank you.

A: Thank you, goodbye.

B: Goodbye.
