# Supplementary material for: Understanding cultural perceptions of sexuality in China and their influence on human papillomavirus vaccine hesitancy
Source: Front Public Health. 2025 Jan 23;12:1462722. doi: 10.3389/fpubh.2024.1462722 (PMC11801254; doi:10.3389/fpubh.2024.1462722)
Supplement: Supplementary file 1 [file Data_Sheet_1.zip › Frontiers_Supplementary_Material/Interview Transcripts - Participant 6.docx]

**Interview Transcripts - Participant 6**

A: Can you share what you know about the HPV vaccine?

B: The HPV vaccine is used to prevent HPV-related diseases. It has been available for many years and comes in three types: bivalent, quadrivalent, and nonavalent, each covering different types of HPV.

A: Do you know what HPV is and how people get infected with it?

B: I don’t know much in-depth information. I always thought these diseases, especially sexually transmitted ones, are related to having an unclean private life or not paying attention to hygiene. But now, it seems like many people are getting infected with HPV.

A: Do you know of any cases among people you know?

B: No, I don’t know anyone personally who has been infected. I’ve seen posts online from people talking about it.

B: Most people around me are students, and we don’t engage in risky behaviors in this regard.

A: Do you take any steps to prevent HPV or cervical cancer?

B: Since I haven’t had any sexual activity yet, I don’t feel that HPV poses a significant threat to me right now. Mainly, I just focus on personal hygiene. I haven’t taken any special precautions yet, but I have thought about getting the vaccine for future prevention.

A: You mentioned considering the vaccine. How hesitant are you on a scale of 1 to 10, with 10 being very hesitant?

B: I’d say around a 6 or 7.

A: What are the main reasons for your hesitation?

B: I’ve already received the bivalent vaccine back in 2017. Now, everyone is talking about the nonavalent vaccine, which offers more protection. But I wonder if it’s necessary to get it so soon.

A: Have you looked into the recommended age range for the nonavalent vaccine? Are you concerned about not needing it at your age?

B: Yes, HPV can affect people of all ages. Cervical cancer is quite common and frightening.

A: Can you explain why you feel it’s too early to get the nonavalent vaccine?

B: The recommended age for the nonavalent vaccine is typically up to the mid-twenties. Recently, I saw on social media that it might be extended to people up to 45 years old. But officially, it’s best for those aged 16-26.

B: I saw that the bivalent vaccine is recommended for ages 9-45, the quadrivalent for 20-45, and the nonavalent for 16-26. I feel like getting it at 26 wouldn’t be too late.

A: Is it because you think it’s too early, or do you want to wait and see? Or are there other concerns?

B: Right now, there’s no urgent need for me to get it. The HPV vaccine is essentially for sexual health, and since I’m not sexually active, I think I can wait until I am. I’m 24 now, so maybe in a few years when I become sexually active, I’ll consider it.

A: Besides thinking it’s too early, are there any other factors contributing to your hesitation?

B: First, I’ve already received the bivalent vaccine, which covers the most critical types of HPV. The nonavalent vaccine covers more types, but the bivalent has already protected me against the most important ones.

B: Secondly, the nonavalent vaccine is quite expensive—around 4000 RMB for the full three doses. That’s a significant financial burden. Lastly, it’s really difficult to get an appointment for the nonavalent vaccine. I’ve tried booking it through a mini-program on WeChat, but it’s always fully booked.

B: Many places now require a lottery system to get an appointment.

B: I even asked at the local clinic where I got my COVID-19 vaccine, and they said it’s very hard to get the nonavalent vaccine because it’s always fully booked.

A: Yes, that's true. Some people even go abroad to get vaccinated.

B: Yes, they go abroad for it. I’ve heard that vaccines abroad are considered better than the ones available domestically. Currently, the vaccines in China are domestically produced, and it's believed that the ones abroad might be more effective. But going abroad takes a lot of time and is quite expensive, significantly increasing the cost.

A: So, you do have concerns about the quality of domestic versus foreign vaccines.

B: Yes, that's right. Also, my mom used to be a nurse. When I got the bivalent vaccine, it was the first HPV vaccine introduced in China, and it was imported, possibly from Hong Kong. My mom and her colleagues, including those at the CDC, have always believed that imported vaccines are better than domestic ones.

A: Are you currently worried because the HPV vaccines in China are now domestically produced?

B: Yes, I am concerned about that. Additionally, I’m unsure if I should get vaccinated now or if it’s necessary to get vaccinated so early.

A: You mentioned earlier that you got the bivalent vaccine for safety and insurance reasons. Have you received any other vaccines, such as the COVID-19 vaccine, for similar reasons?

B: Everyone has had the COVID-19 vaccine. I remember the school kept urging us to get it.

A: For the later doses, some might have been optional, like the third dose.

B: Yes. Initially, my family was reluctant to get vaccinated, thinking the vaccines were risky, especially the COVID-19 vaccine. But my mom's workplace mandated it, so she got the three-dose vaccine. The doctor who administered it said it was good. We found out our local clinic occasionally had the three-dose vaccine, so we registered and got it when it became available.

B: Initially, we thought the vaccine might be dangerous, but after hearing from healthcare professionals and due to the mandate, we changed our minds.

A: You mentioned the second reason for hesitation is the cost. Do you know the exact prices of the HPV vaccines?

B: The nonavalent vaccine costs about 1300 RMB per dose, the quadrivalent around 800 RMB, and the bivalent about 400 RMB. Altogether, the nonavalent vaccine costs nearly 4000 RMB for three doses, which is expensive, especially if you can’t book it yourself and need to use a booking service.

A: Have you ever incurred significant medical expenses?

B: I haven't had any major illnesses as an adult, just minor ones. Another concern I have with vaccines stems from my experience with the COVID-19 vaccine. After contracting COVID-19 in December 2022, I experienced lingering heart issues. When researching online, I found many reports suggesting these long-term effects could be linked to the COVID-19 vaccine. A friend of mine developed severe muscle weakness after the vaccine and was questioned by doctors if he had received it, as many patients with similar issues had.

A: So, the side effects of the COVID-19 vaccine have made you hesitant about the nonavalent HPV vaccine?

B: Yes, it has made me cautious about all future vaccines. I’d prefer to wait until the vaccines are more mature and proven to be safer.

A: Are you worried more about potential known side effects of the nonavalent vaccine, or is it more about a general fear of possible side effects?

B: It’s more about a general fear of potential side effects.

A: You mentioned waiting until the vaccine is more mature. Does that mean you are currently in a wait-and-see phase?

B: Yes, I prefer to wait and see.

A: Let's first discuss the issue of cost. What do you think is worth spending 5000 RMB on in general, especially in terms of medical expenses?

B: I think if you're sick and need to go to the hospital, any amount of money is justified. As for vaccines, since the nonavalent HPV vaccine has alternatives like the bivalent and quadrivalent vaccines, it’s a different story.

A: You mentioned you’ve already received the bivalent vaccine because the nonavalent wasn’t available at the time. If it had been, you would have preferred to get it, right?

B: Yes, I received the bivalent vaccine before the nonavalent was available. If the nonavalent had been an option, I would have chosen it to be fully covered.

A: You brought up the topic of HPV and sexual activity. What do you think is the connection between them?

B: I believe most HPV infections are related to sexual activity. It could be due to personal issues or issues with a male partner, similar to how HIV is transmitted sexually.

A: Besides the reasons you've mentioned, you indicated that your vaccine hesitancy isn't very strong. What factors might motivate you to get the HPV vaccine in the future?

B: The primary factor would be if I perceive a high risk of contracting HPV, such as becoming sexually active, especially with multiple partners. That would make the vaccine essential. Another factor would be if the nonavalent vaccine becomes more widely available and easier to obtain.

A: Have you found any information about HPV online?

B: Yes, almost all my information about HPV comes from the internet, including what HPV is, how people get infected, treatment options, vaccine prices, and how to book appointments.

A: So, you haven't heard much about the HPV vaccine from offline sources?

B: No, discussions about HPV are rare offline. Unless you visit a community health center for a vaccination, you don't see much information about HPV.

A: Have you discussed HPV-related information with your classmates or friends?

B: Yes, but these discussions are infrequent. Mostly, we talk about whether to get the vaccine and how to book an appointment. One of my classmates had to resort to a booking service in Beijing and ended up getting scammed. So, if you can't book an appointment yourself, it's not worth using such services.

A: Do they generally support getting the vaccine, have reservations, or oppose it?

B: Most of my friends are undecided. They find it difficult to book and consider it expensive. If they manage to book an appointment, they’ll get vaccinated; if not, they let it go. That’s the general attitude.

A: What are their main concerns?

B: We haven't had in-depth discussions. Mostly, it's about the difficulty in booking and the cost. Sometimes, there’s a lack of parental support for the expense. We don't often talk about the reasons for getting the HPV vaccine in relation to sexual activity.

B: It’s not easy to discuss these topics with people you’re not very close to.

A: How does your family feel about you getting the nonavalent vaccine, or how did they feel about the bivalent vaccine?

B: When I got the bivalent vaccine, it was right after high school graduation, and I didn’t know much about it. My mom is very supportive of vaccinations. She believes that since cervical cancer is quite common among women, getting vaccinated is important. She felt I was at the right age for it. As for the nonavalent vaccine, I’ve discussed it with her, and she thinks it’s fine either way since I already have the bivalent vaccine. But I believe the nonavalent vaccine offers broader protection.

A: Let's revisit our earlier discussion about online information. Do you generally find the information about HPV vaccines on the internet to be positive, negative, or neutral?

B: I think the information about HPV vaccines is mostly positive. The promotion encourages people to get vaccinated. However, I do come across negative information, which makes me hesitant. The positive messages persuade me to get vaccinated, while the negative ones discourage me.

A: So, you're weighing the positive and negative information as you make your decision.

B: Yes.

B: Besides the negative factors, another reason for my hesitation is that I've already had the bivalent vaccine. Additionally, the cost and difficulty of getting an appointment are concerns.

A: Yes, and when you got the bivalent vaccine, you mentioned that your arm was swollen and sore for about a week. Although the COVID vaccine didn't have that effect on you, this negative experience has left you with some reservations about getting the nonavalent vaccine, especially since others have reported similar side effects.

B: Exactly.

A: That concludes our interview. Thank you very much for your participation and support.

B: Ok, no problem.
