# Supplementary material for: Understanding cultural perceptions of sexuality in China and their influence on human papillomavirus vaccine hesitancy
Source: Front Public Health. 2025 Jan 23;12:1462722. doi: 10.3389/fpubh.2024.1462722 (PMC11801254; doi:10.3389/fpubh.2024.1462722)
Supplement: Supplementary file 1 [file Data_Sheet_1.zip › Frontiers_Supplementary_Material/Interview Transcripts - Participant 7.docx]

**Interview Transcripts - Participant 7**

A: What information do you currently know about the HPV vaccine?

B: I first learned about HPV in college. Our class representative mentioned that the campus clinic offers the vaccine, but only the bivalent and quadrivalent types. I found out that it can prevent certain diseases in women, like cervical cancer and some types of warts.

A: Do you know why people get infected with HPV?

B: Not really. Maybe it has to do with personal hygiene or one's private life.

A: So, you haven't specifically taken measures to prevent HPV infection?

B: No, I haven't.

A: On a scale of 1 to 10, how hesitant are you about getting the vaccine?

B: I haven't been vaccinated.

A: How would you rate your level of concern or hesitation about the HPV vaccine?

B: I'd say about a 6.

A: What are your main concerns about the HPV vaccine?

B: If I were to get vaccinated, I would choose the nonavalent vaccine. It requires multiple doses and is quite expensive. I'm also worried about potential side effects. Plus, I've read that it doesn't prevent all types of HPV, just the strains listed on the vaccine.

A: Besides these concerns, could your hesitation be influenced by personal beliefs, your understanding of vaccines, or trust in the healthcare system? Or perhaps societal factors like cultural views on discussing sexual health?

B: No, those aren't factors for me.

A: You don't have any concerns about discussing these topics?

B: I just feel that I'm unlikely to contract HPV, so I haven't looked into it much.

A: When you say you're unlikely to contract HPV, is it because no one around you has had it, or because you don't engage in activities that put you at risk, like sexual contact?

B: Yes, no one I know has had it, and I don't engage in risky behaviors.

A: Considering all these factors, you mentioned cost earlier. Can you explain your thought process regarding the expense? For example, some people might find it essential to get a rabies vaccine despite the cost because of the high risk if they don't. Can you discuss your reasoning in terms of cost?

B: I just think I'm unlikely to need it, so I can't justify the expense.

A: You also mentioned concerns about side effects. Do you know what specific side effects the HPV vaccine might cause?

B: No, I don't.

A: You just know there could be side effects?

B: Yes, I think any vaccine or injection might cause some side effects.

A: Is this concern influenced by previous vaccine experiences? For instance, when getting the COVID-19 vaccine, people were often asked to stay for observation in case of side effects. Did you have similar concerns with the COVID-19 vaccine?

B: Yes, but the COVID-19 vaccine was mandatory at my school, so I didn't have a choice.

A: You mentioned your level of concern is a 6 out of 10, meaning you wouldn't completely rule out getting the vaccine. What would encourage you to get the HPV vaccine in the future?

B: First, if I could afford it. Also, if I learned more about HPV and realized that the risk of infection is high.

A: Have you ever discussed this topic with classmates or friends in person?

B: No, I haven't.

A: Do you have any friends who have received the vaccine?

B: Yes, one classmate mentioned it. He said it was hard to get an appointment, and he ended up getting vaccinated in Macau.

B: He had to take leave every time he went to Macau for the shots, which seemed very inconvenient to me.

B: Other classmates haven't really talked about it, and most haven't gotten vaccinated either. Among my roommates, none of us have been vaccinated, and I'm not sure about others.

A: Have they ever shared their concerns with you, or did they just not get vaccinated?

B: No, we don't discuss this topic.

A: Besides talking to friends or classmates, does your family know about this vaccine? Do they discuss it with you?

B: My parents aren't very familiar with the HPV vaccine. I mentioned it to them once, but they didn't understand what it was.

A: After realizing they didn't understand, did you try to explain it to them, or did you just leave it at that?

B: I didn't feel the need to explain it in detail.

A: I see. You felt it would be difficult to explain, so you didn't.

B: Yes.

A: You mentioned earlier that you mainly get information online. Is that correct?

B: Yes.

A: When you look online, do you see more positive or negative information about the vaccine?

B: Mostly positive. The information encourages people to get vaccinated, emphasizing the importance of the vaccine.

A: Have you come across any negative news?

B: No.

A: If you had a deeper understanding of the vaccine, would you consider educating others about it?

B: Maybe after I've been vaccinated myself and experienced its benefits, I would feel more confident about recommending it to others.

A: One last question: Do you think there's a connection between sexual activity and HPV?

B: Yes, you mentioned earlier that poor personal hygiene or unprotected sex can lead to HPV. If a partner is not clean, it's easy to contract such infections.

A: That concludes our interview. Thank you very much for your time.

B: You're welcome.
