# Supplementary material for: Understanding cultural perceptions of sexuality in China and their influence on human papillomavirus vaccine hesitancy
Source: Front Public Health. 2025 Jan 23;12:1462722. doi: 10.3389/fpubh.2024.1462722 (PMC11801254; doi:10.3389/fpubh.2024.1462722)
Supplement: Supplementary file 1 [file Data_Sheet_1.zip › Frontiers_Supplementary_Material/Interview Transcripts - Participant 8.docx]

**Interview Transcripts - Participant 8**

A: What information do you have about the HPV vaccine?

B: Honestly, I don't know much. I remember this topic being quite popular a year or two ago, and many of my classmates were getting the vaccine. They said it helps prevent some diseases, but I wasn't very clear on the specifics. I just thought, like with any vaccine, it has both pros and cons. I didn't see the need to get it, similar to how I felt about the COVID-19 vaccine.

A: So you don't know exactly what HPV is, or how it spreads?

B: I think if university students often travel, things like hotel bedsheets or toilets could be sources of infection.

A: Are there other ways it can spread?

B: It's not transmitted through saliva. So, it might be more likely to spread in public places.

A: Have you specifically looked up how HPV spreads or how to prevent infection?

B: No, I haven't.

A: How likely are you to get the vaccine, on a scale of 1 to 10?

B: Probably around a 5.

A: So you're leaning towards not getting vaccinated and have significant hesitations?

B: Yes, because I'm not sure about its effectiveness. Vaccines are not always 100% effective, and I think it might have some negative effects on my body. So, I'm not very inclined to get it.

A: You mentioned you don't know much about HPV. It primarily spreads through sexual contact in addition to public hygiene issues. Are you aware of this?

B: Yes, I'm aware of that aspect.

A: Did you learn this from news or online posts?

B: Yes, especially on TikTok. Information spreads quickly and widely there, so I often see it mentioned.

A: So you've seen educational content about HPV on TikTok?

B: Yes.

A: Can you elaborate on your concerns about the HPV vaccine? You can list them as points if you like.

B: First, I'm not convinced it is 100% effective, and it might harm my body. Second, it might cause adverse reactions after the injection. Third, the vaccine has a limited duration of effectiveness; it doesn't provide lifelong protection.

A: Are there other factors, like cost? Do you think the vaccine is too expensive?

B: Yes, cost is a concern. It can be quite expensive, around 7,000 to 8,000 RMB for three doses. It's about three doses, costing around 4,000 to 5,000 RMB. There might be additional costs depending on where you get it. And it's also hard to get an appointment online.

A: Are there any personal concerns about vaccine safety that you've mentioned? Do you also have any distrust towards the healthcare system or medical professionals?

B: Yes, that's also a factor. I have classmates whose family members are doctors, and they also believe that getting this vaccine might not be necessary or beneficial. So, hearing these opinions from healthcare professionals also influences my decision.

A: These are internal discussions among doctors, suggesting that they don't see the necessity, and that has a significant impact on you, right?

B: Exactly, because when you decide to get vaccinated, you usually consult a doctor. If some doctors believe it's unnecessary, it certainly affects my decision-making.

A: Besides personal factors, do you think there are societal influences, such as traditional beliefs, that might discourage discussing HPV, especially since it's primarily transmitted through sexual activity?

B: Yes, but I haven't encountered much resistance in discussing it among people I know.

A: So, it's not a big concern when talking about it with people around you?

B: Right, it seems like people around me don't bring it up often.

A: Earlier, you mentioned concerns about adverse reactions as one of your main reasons for hesitating about the vaccine. Do you know what specific adverse reactions it might cause?

B: It's possible that friends who've been vaccinated might post on social media about experiencing pain at the injection site and feeling weak for a period afterward.

A: You rated yourself a 5 in terms of likelihood to get vaccinated, which means you're not completely opposed to getting vaccinated, correct?

B: Yes, there's also some physical discomfort to consider, but I'm not ruling out getting vaccinated someday.

A: What factors do you think would most likely prompt you to register and receive the HPV vaccine in the future? Or are there any specific factors?

B: Perhaps when I'm economically independent after finishing school, that might be a critical point for me. When I have more financial stability, I might prioritize my health more and follow the trend of others getting vaccinated.

A: Are there other considerations, like waiting for more mature technology, maybe five or ten years from now?

B: Not that long, maybe just a couple of years. HPV vaccinations are generally recommended before a certain age, so waiting too long might not be ideal. Also, it seems like the hype around it has diminished recently. It was more talked about a year or two ago, but now, I hardly see it on TikTok.

A: I feel the same way. It was quite popular a few years ago, but it seems like there's less promotion now. Weren't a lot of people talking about getting vaccinated back then? Did you feel the urge to schedule an appointment and join them?

B: No, because, similar to how I felt about the COVID-19 vaccine, I have an inherent hesitation about getting vaccinated. Also, TikTok's development hasn't been as good in recent years, and the spread of information there is very fast, making it hard to distinguish between truth and falsehood. When something becomes trendy and everyone starts posting about it, negative reviews might get drowned out, and it seems like everyone is urging others to get vaccinated. I think there's more of a herd mentality involved, where many people may not fully understand but think it's good to get vaccinated for prevention.

A: Indeed, after the hype, many people around me directly made appointments, and when I asked them, they didn't really understand the details either.

A: You mentioned earlier that during that time, a classmate of yours went to Hong Kong specifically to get vaccinated. Could you elaborate on that?

B: Yes, during my junior or senior year of high school, which was about three or four years ago, HPV vaccination wasn't as widespread in mainland China as it is now. So, my classmate traveled to Hong Kong multiple times to get vaccinated.

A: Was it because he had to get multiple shots, or was it due to other reasons?

B: Yes, he had to travel back and forth because of that. At that time, he had connections in Hong Kong, so he managed to secure appointments for the vaccinations. Otherwise, it would have been difficult for him to get vaccinated.

A: So, it was through his connections in Hong Kong that he managed to schedule the appointments?

B: Yes, exactly. So, it was clear that having connections was necessary to secure those vaccinations.

A: Right, without those connections, it would have been difficult to get vaccinated.

B: Yes, these situations tend to favor those who are more affluent.

A: Earlier, you mentioned that you've been afraid of needles since childhood, and recent events like the COVID-19 vaccinations have also had an impact on you. Could you elaborate on that?

B: Yes, in recent years, events like COVID-19 have had a significant impact. I'm not exactly afraid of getting injections myself, but I believe that injections inherently have negative effects on the body. It's unlikely that they are entirely beneficial, and everyone's physical condition varies. What might have no effect on one person could cause different reactions in another.

A: Apart from the factors we discussed earlier, such as waiting until after starting work, are there any other factors that might influence your decision, such as considerations related to sexual activity?

B: Yes, inevitably, as I get older in a few years, these considerations will become unavoidable. Everyone engages in sexual activity. Of course, it's also for my own health and the health of my partner.

A: Do you think there's a connection between sexual activity and HPV?

B: Yes, sexual activity is one of the transmission routes, and it could lead to bacterial infections, potentially exacerbating the risks of viral infection.

A: Besides these aspects, have you discussed HPV vaccination with friends or classmates in person? Could you elaborate on that?

B: Not really. Among friends, there hasn't been much discussion about it. Usually, it's just about who managed to get an appointment or who had reactions after getting vaccinated.

A: Do you think this approach is somewhat blind? We don't really discuss what this virus is, how it's transmitted, but rather, everyone rushes to discuss whether they got the HPV vaccine, how it felt, and whether they experienced discomfort.

B: I think it's largely a herd mentality. TikTok seems to have become the primary channel for everyone to gather information these days. So, when something becomes popular on TikTok, many people tend to follow suit, similar to how influencers promote products. If a top influencer says something is good and many people buy it, everyone else tends to follow suit. However, many times, the product may not actually be suitable for everyone.

A: Why haven't you discussed with others what this virus is and why it's important to get vaccinated?

B: Because I myself may not know much about it. I'm not familiar with this area, so discussions among friends about this topic are relatively rare. More often, discussions among friends are about things like where to go for outings rather than about this topic.

A: When discussing this topic with others, do they tend to support vaccination, mention strong side effects, or oppose it?

B: They don't seem to express strong opinions. If they decide to get vaccinated, they share their experiences, but they don't try to convince me whether or not to get vaccinated.

A: Besides classmates and friends, have you discussed these matters with your family?

B: My parents, perhaps due to their lower educational background, haven't brought up this topic with me either. They probably don't understand much about it.

A: So, you haven't mentioned to them that you're considering getting the 9-valent or 4-valent HPV vaccines?

B: Right. I'm quite independent, so I tend to handle things like this on my own after achieving financial independence.

A: You mentioned relying more on online sources like TikTok, Xiaohongshu, and possibly WeChat for information. Is that correct?

B: Yes, there's a bit of a generation gap with my parents. If I had siblings closer to my age, we might communicate better. So, the internet is where I primarily get information.

A: Are TikTok and Xiaohongshu your main platforms, or are there others?

B: Those are the main ones, including articles shared on WeChat.

A: What types of articles do you usually come across?

B: They usually include HPV vaccine explanations, discussions about its potential harms, and personal experiences shared by others.

A: Among these information sources, do you notice more positive or negative perspectives?

B: Currently, there seems to be a predominance of positive views. Negative opinions exist but seem overshadowed, similar to what happened with the COVID-19 vaccine. Some people speak negatively about it, but overall, positive views prevail.

A: Lastly, I'd like to touch upon cultural constraints.

B: Yes, among friends, it's rare to discuss such topics. It's almost taboo. Even if someone is in a relationship, friends might ask superficially about it, but asking directly about sexual activity or whether you're considering HPV vaccination is uncomfortable for everyone. Few people would bring it up.

A: Right, so in this context, it's challenging for me as an interviewer to focus on these points, especially since we're not familiar and discussing it with friends is also awkward.

B: Yes, even among friends, this topic rarely comes up.

A: Exactly. As an interviewer, focusing on these points might feel uncomfortable for both of us.

B: Yes, especially for girls, they might be more reserved. Discussing such aspects of personal life can be embarrassing.

A: Do you think there's a tendency for vaccines to be stigmatized in society? For instance, some people argue that practicing abstinence and maintaining personal hygiene can suffice without needing vaccines. Do you feel this viewpoint influences you?

B: Yes, I think older generations, like our parents, often hold more conservative views like that. But with the advancements in our era, I believe it's not just about promiscuity. Even in committed relationships, infections can occur due to various factors. Many adults tend to unfairly label girls as impure, which reflects a significant bias against women in our society.

A: I understand. I've come across similar perspectives on platforms like Zhihu, where people question why girls would need the vaccine if they lead normal lives. It does seem to stem from a considerable amount of bias.

B: Yes, there's a lot of hostility towards women in many aspects.

A: Indeed. Even getting a vaccine can be metaphorically interpreted negatively, right?

B: Exactly, it's just a preventive measure. For example, when staying at hotels, you can't be sure if the previous guest was clean or not. If you contract an infection unknowingly, society might still blame you for being promiscuous. The bias against women is really pervasive.

A: Absolutely. Alright, I think that concludes our interview here. Thank you.
