# Supplementary material for: Understanding cultural perceptions of sexuality in China and their influence on human papillomavirus vaccine hesitancy
Source: Front Public Health. 2025 Jan 23;12:1462722. doi: 10.3389/fpubh.2024.1462722 (PMC11801254; doi:10.3389/fpubh.2024.1462722)
Supplement: Supplementary file 1 [file Data_Sheet_1.zip › Frontiers_Supplementary_Material/Interview Transcripts - Participant 9.docx]

**Interview Transcripts - Participant 9**

A: Could you discuss what information you know about the HPV vaccine?

B: Recently, I know it can prevent cervical cancer. There are different types like 2-valent, 4-valent, and 9-valent vaccines, each targeting different strains. The 9-valent one provides the most comprehensive protection. Also, I understand getting vaccinated doesn't guarantee complete immunity against cervical cancer. I've also seen news about the age range for vaccination expanding to 9-45 years, and I keep up with the latest policies and products related to this.

A: You seem to have a comprehensive understanding.

B: Including how it used to be difficult to schedule appointments, that's about it.

A: Do you know about the transmission pathways of HPV, or why one might get infected?

B: I've heard that having a clean personal life lowers risks. Common places like public pools can also spread HPV. If precautions aren't taken, it's possible to contract it.

A: So you're aware of these transmission paths. Does knowing them influence your decision to actively prevent HPV infection?

B: Everyone considers their health, especially women who focus on reproductive health. There are concerns about various gynecological issues, which can be quite frightening.

A: Based on your knowledge of the HPV vaccine, how hesitant are you currently about getting vaccinated, on a scale of 1 to 10?

B: Maybe an 8.

A: That's quite high.

B: Yes.

A: Let's discuss specifically what factors contribute to your hesitation.

B: Alright. First, most discussions about the HPV vaccine tend towards the 9-valent one. I didn't even know about it until about three or four years ago when I started my graduate studies. When I first found out, it was notoriously difficult to schedule, and people around me who tried had to wait a long time. The whole process seemed cumbersome, and personally, I dislike waiting. Also, because the 9-valent vaccine is hard to schedule, online information makes it seem like a rare commodity that everyone rushes to get, which I find unsettling. I'm somewhat rebellious against trends and the excessive glorification online. Moreover, the government hasn't reduced costs or promoted widespread availability, which seems odd. Overall, these factors contribute to my hesitation.

B: Following up on what I just mentioned, I feel the internet has somewhat sensationalized the HPV vaccine. There's concern it might foster a black market—like scalpers reselling at inflated prices—and it's hard to secure appointments. It's almost like a frenzy, akin to scarcity marketing. I worry there might be gray market activities or exploitation by criminals profiting from consumers' concerns, potentially infringing on their rights. Additionally, there was a preference for imported vaccines when domestic options like 2-valent and 4-valent weren't widely available. This led to conspiracy theories suggesting foreign interests wanted us dependent on these vaccines, possibly due to adverse effects they carry post-vaccination, which might harm women's health. I've encountered these theories and they contribute to my concerns. Another factor is the vaccine's side effects. Although not everyone experiences them, there are reports of menstrual irregularities or polycystic ovary syndrome after vaccination, which worries me. I'm unsure if these effects are reversible or might persist, given my uncertain physical constitution. Furthermore, based on my biological and medical studies, I understand HPV doesn't always progress to cervical cancer; our immune systems can often clear it. This belief in bodily resilience combined with regular check-ups minimizes my perceived risk, leading to some sense of complacency. Regarding costs, while I'm unsure if they're covered by medical insurance, I've seen reports suggesting it might be covered, making it acceptable. However, others might find the cost prohibitive. Lastly, reading conflicting opinions on platforms like Zhihu, especially negative ones from supposed medical professionals, has also influenced me negatively, planting seeds of doubt early on.

A: Yes, especially on Zhihu, there were numerous posts advocating for HPV vaccination around 2017-2018. It felt like a herd mentality. Have you encountered similar examples?

B: Yes, many friends were caught up in the hype. Once someone started booking appointments, everyone followed suit without necessarily researching thoroughly or making informed decisions.

A: It seems like many people may not fully understand what the HPV vaccine is for. Some might not even distinguish between the different types like 2-valent, 4-valent, or 9-valent—they just go ahead and book appointments.

B: Yeah, they might write off HPV as having no significant impact and simply act out of fear, without really understanding much about it, just following the trend.

B: You might say I have a contrarian streak—I tend to hesitate a lot before making decisions, especially with both big and small choices. So, I prefer to gather comprehensive information from various sources before deciding on anything. It's also influenced by personal tendencies, I believe.

B: Right, and navigating online information can be quite chaotic. Some say you absolutely must get vaccinated, while others warn against it. Platforms like Zhihu especially—I've heard stories where healthcare professionals anonymously advise against it.

A: Exactly, the more you're exposed to this mishmash of opinions, the more hesitant you become in making a decision.

B: Indeed, I find myself affected by negative opinions, which makes me even more cautious.

A: Especially when those negative views, the ones you don't want to hear, start surfacing. It undermines your confidence in the decision.

B: Yeah, and sometimes these negative factors overshadow the positive ones, leading you to reconsider getting vaccinated altogether. I'm not really into conspiracy theories. I might inadvertently absorb some political viewpoints when reading, but I don't actively seek out that kind of content, nor dwell on it too much.

B: Yeah, and as you mentioned earlier about the conspiracy theories, it's also a point of interest. And what you said about the mentality of relying on luck earlier, and you said, indeed, from 9 to 45 years old, it seems that the news last year, or September, I'm not too clear about that. So, before this news came out, everyone didn't know that it could go up to 45 years old, right? But I feel that there is a trust in medical technology, and there may be better and more universal vaccines developed in the future, specifically for a wider audience of women. So, I think there's a wait-and-see attitude, that I just can't make a decision and so on.

B: Hoping for the future development of vaccine technology.

A: Yeah, and now you don't really have to rush to get the 9-valent vaccine. It's actually quite easy to schedule now. Yeah, the changes in the world are happening quite fast.

B: Yeah, there were a lot of people who went to Hong Kong and Macau recently.

B: Yeah, and some even flew abroad just to get vaccinated. Hong Kong and Macau are close enough, but some went even further, which I find quite unbelievable. It's really quite unbelievable.

B: Someone asked if you still need to get three shots of the 9-valent HPV vaccine. I heard from interviews that some classmates went to Macau to get vaccinated, took leave each time, and ran over to get vaccinated. That was during the pandemic, and they went to Macau three times, which seems a bit extreme.

B: In terms of the mentality of relying on luck, have you seen anyone around you who holds such views, including on the internet?

A: So, does your sense of invulnerability come from thinking, "I'm not likely to get HPV, I'm not likely to be infected with HPV"?

B: On one hand, I definitely have a general understanding. As I mentioned earlier, most of the time, one's immune system can clear it out by itself. The probability of it developing into cancer isn't very high. For instance, I had HPV before, but back then I wasn't sexually active and it was just a type that appeared on my foot. It looked like a wart, and later it was frozen off. It didn't have much impact at all. So, if you go by what doctors say, having HPV doesn't have a big impact, and the chance of it developing into high-risk HPV, let alone cervical cancer, is very small. So, I don't find it that frightening.

B: It's not all about a sense of invulnerability; it's that the probability itself is quite low. Moreover, for some low-risk types of HPV, there's really no need to worry too much. Some simple treatments suffice; it's not as serious as some might think. Like some low-risk types that might cause warts on hands. I had it on my foot, and later I had it frozen off a few times. It's something that high school and college students might contract. I got it in my sophomore year, and at the time, I probably didn't know why—maybe from wearing shoes or something. When I asked the doctor why I got HPV when I hadn't been sexually active, it seemed odd to me. But later, the treatment wasn't as mysterious as I thought; it was quite straightforward, and overall, it was relatively easy.

B: It's not as scary as what's sometimes said online, that you absolutely must get vaccinated or face dire consequences. Maybe because I had symptoms of low-risk types myself, and there are so many types of HPV, so I don't think it's that scary.

A: If you were to consider getting vaccinated in the future, what factors would prompt you to do so?

B: Maybe if there were some abnormalities in my gynecological exams. Currently, there don't seem to be any potential risks, and in places like public swimming pools or communal baths, I don't think I'd be likely to go. Yeah, maybe I haven't encountered it yet, and the future is still uncertain.

B: Currently, there's a lot of hesitation, meaning I'm quite hesitant and wouldn't go for the vaccine.

A: Right, and I think maybe people's views can vary greatly at different times, so my thoughts might change suddenly after this period. So, it's quite fascinating.

B: Perhaps one day you'll suddenly decide to get vaccinated. Are you not opposed to it if there's a genuine need? Right now, you seem to be in a wait-and-see mode, not feeling pressured to book an appointment or get vaccinated immediately. It's quite neutral; there's no strong urge to do it.

A: Besides moments of hesitation and potential turning points, like we just discussed about online channels, have you talked about HPV vaccines offline with friends or classmates?

B: I feel like I've talked more with my mom about it. She might mention how the neighbor's daughter got vaccinated. Surprisingly, my classmates haven't really talked about it much. They might say as long as you focus on your own sexual health, there shouldn't be much of a problem. That's how they put it. It seems to be more of a family discussion topic. My mom was urging me to make an appointment and get vaccinated quickly, but I have my own thoughts on the matter, so I haven't pursued it further.

A: Do you ever initiate conversations with others about HPV vaccines or infections?

B: No, I wouldn't. Unless someone has concerns in that area or if they feel they might be infected, maybe they'd talk about it. I don't think I'd actively bring up topics like HPV and so on. You know? Compared to discussing preventive measures or saying that the body has a good chance of fighting these things off, it seems they don't really care. It's like, "I'll get vaccinated first and then think about it." They haven't really deeply considered what the vaccine actually prevents.

A: Are there certain taboos or conservative attitudes that make some topics off-limits? Because HPV infections involve elements of sexual activity, does it affect you?

B: I think it depends on the relationship. With roommates, we sometimes talk about it casually. It's possible to discuss, but with general acquaintances, like classmates you just see in class or pass by, and with whom you have limited interaction offline, you wouldn't bring it up. It's talked about less. With friends, though, if you're interested, it's okay to discuss it, I think. Yeah. For example, some friends might bring up how older generations might not understand, and there might even be some moral judgment. Like, getting vaccinated implies moral or promiscuous behavior and deserves punishment. This kind of thinking shouldn't exist among today's college students. Or does it? Would you be influenced by this kind of thinking? It's a bit odd to bring up this example, but I feel like it fits. When I was in my second year of graduate school, I moved dorms once, from one building to another. We got to redecorate. In our first year, there were five of us living together, but when we moved to the new building, there were only four of us. One of our former roommates had already completed the 9-valent vaccine during our first year, and I felt like he had a "I'm protected now, so I can do whatever I want" attitude. It seemed like his personal life was quite chaotic. It's like he thought, "Now that I've been vaccinated, I'm safe, so I can do whatever." It's quite fitting with what you mentioned earlier. Some people do have such thoughts. I've read about it online before, and this was the first time I actually encountered such a situation. But this phenomenon does exist and it's around you.
